# Testing for the Markov property in time series via deep conditional generative learning

**DOI:** 10.1093/jrsssb/qkad064

**Published:** 2023-06-23

**Authors:** Yunzhe Zhou, Chengchun Shi, Lexin Li, Qiwei Yao

**Affiliations:** Division of Biostatistics, University of California at Berkeley, Berkeley, CA, USA; London School of Economics and Political Science, London, UK; Division of Biostatistics, University of California at Berkeley, Berkeley, CA, USA; London School of Economics and Political Science, London, UK

**Keywords:** deep conditional generative learning, high-dimensional time series, hypothesis testing, Markov property, mixture density network

## Abstract

The Markov property is widely imposed in analysis of time series data. Correspondingly, testing the Markov property, and relatedly, inferring the order of a Markov model, are of paramount importance. In this article, we propose a nonparametric test for the Markov property in high-dimensional time series via deep conditional generative learning. We also apply the test sequentially to determine the order of the Markov model. We show that the test controls the type-I error asymptotically, and has the power approaching one. Our proposal makes novel contributions in several ways. We utilise and extend state-of-the-art deep generative learning to estimate the conditional density functions, and establish a sharp upper bound on the approximation error of the estimators. We derive a doubly robust test statistic, which employs a nonparametric estimation but achieves a parametric convergence rate. We further adopt sample splitting and cross-fitting to minimise the conditions required to ensure the consistency of the test. We demonstrate the efficacy of the test through both simulations and the three data applications.

## 1 Introduction

The Markov property is fundamental and is commonly imposed in time series analysis. For instance, in economics and reinforcement learning, the Markov property is the foundation of the Markov decision process that provides a general framework for modelling sequential decision making. In finance and marketing, the Markov property is widely assumed in most continuous time modelling. See Chen and Hong (2012) for a review. Correspondingly, testing the Markov property, and relatedly, inferring the order of a Markov model, are of paramount importance in a broad range of applications.

Such a testing problem, however, is highly nontrivial and poses many challenges, especially for high-dimensional time series. For the Markov property test, Aït-Sahalia (1997) proposed a nonparametric test based on the Chapman–Kolmogorov equation and smoothing kernels. Chen and Hong (2012) tackled the testing problem based on the conditional characteristic function (CCF) estimated by local polynomial regressions (LPRs). However, kernel smoothers, including LPRs, suffer from a poor estimation accuracy in moderate to high-dimensional settings, leading to an inflated type-I error or a low power for the tests. For the order determination in nonparametric autoregression, Cheng and Tong (1992), Yao and Tong (1994) and Vieu (1995) developed some cross-validation based methods, and Auestad and Tjøstheim (1990) and Tschernig and Yang (2000) proposed a final prediction error based criterion. But none of those order determination methods are based on hypothesis testing, and they all assume the dimension of the time series is fixed. More recently, Shi et al. (2020) developed a quantile random forest algorithm and a doubly robust procedure to test the Markov assumption in the context of reinforcement learning. But their method, as we show later in Section 5, would fail to control the type-I error in the time series setting.

In this article, we propose a nonparametric testing procedure for the Markov property in high-dimensional time series via deep conditional generative learning. The proposed test can be sequentially applied for order selection of the Markov model as well. Our proposal makes unique and useful contributions in several ways.

Particularly, we utilise some state-of-the-art deep conditional generative learning methods to address a classical yet challenging statistical inference problem in time series analysis. Deep conditional generative models include mixture density networks (MDNs) (Bishop, 1994), conditional generative adversarial networks (Mirza & Osindero, 2014), conditional variational autoencoders (Sohn et al., 2015), and normalising flow models (Kobyzev et al., 2020). They provide a powerful set of tools to flexibly learn conditional probability distributions, and have been used in numerous applications, such as computer vision, imaging processing, and artificial intelligence (Jo et al., 2021; Shu et al., 2017; Wang et al., 2018; Yan et al., 2016). Nevertheless, these tools are much less used and studied in the statistics literature. We employ this family of models to learn highly complex conditional distributions in a nonparametric fashion, and demonstrate their advantages over the more traditional kernel smoothers including LPRs, especially in a high-dimensional setting.

Meanwhile, it is far from a simple application of some ready-to-use deep learning tools, but instead it requires both crucial modification of the methods and careful characterisation of their theoretical properties. We build our testing procedure based upon MDNs (Bishop, 1994), combined with several crucial new components. First, we propose a new MDN architecture to model the conditional distribution of a multivariate response. Based on such an architecture, we learn two distributional generators, a forward generator and a backward generator, then properly integrate the two generators to construct the test statistic. Second, we derive the convergence rate of the MDN estimator in Theorem 3 , which is crucial to establish the consistency of our proposed test, but is not currently available in the MDN literature. In particular, we provide a sharp upper bound on the approximation error of MDN in Lemma 1 when the underlying conditional density function follows an infinite conditional Gaussian mixture model. We remark that, although it is possible to obtain a bound by directly applying Lemma 1 of Barron (1993), it would only yield a very loose bound; see Section 4.1 for more details. To our knowledge, we are among the first to systematically study the error bound of MDN, and our results are useful for the general theory of deep (generative) learning methods (see e.g. Chen et al., 2020; Farrell et al., 2021; Liang, 2021; Zhou, Jiao et al., 2022; Zhou, Su et al., 2022). Third, we show the proposed test controls the type-I error in Theorem 5, and has the power approaching one in Theorem 6. We show that our test statistic achieves a parametric convergence rate and a parametric power guarantee while its components are estimated nonparametrically. This is made possible because the way in which we combine the two distribution generators yields a doubly robust estimator of the test statistic (Tsiatis, 2007). Thanks to this double robustness, the bias of our test statistic estimator decays to zero faster than the rate of the individual nonparametric distribution generator. Finally, to avoid the requirement of certain metric entropy conditions for the distribution generator estimators (Chernozhukov et al., 2018, Equation (1.6)), we further employ the sample splitting and cross-fitting strategy (Romano & DiCiccio, 2019) to ensure the size control of the test.

The rest of the article is organised as follows. We formulate the hypotheses and propose a doubly robust test statistic in Section 2. We develop the corresponding test, as well as a forward sequential procedure for order determination in Section 3. We establish the theoretical guarantees in Section 4. We carry out simulations in Section 5, and illustrate with three real datasets in Section 6. We relegate all technical proofs to the Online Supplementary Material, Appendix.

## 2 Hypotheses and test statistic

### 2.1 Hypotheses

We first formulate the hypotheses of interest. Consider a strictly stationary *d*-dimensional time series, $X_{t}=(X_{t,1},X_{t,2},\ldots ,X_{t,d}{)}^{⊤}$, t≥1. We target the following pair of hypotheses:


(1) $$\begin{array}{cc} & H_{0}\;:\;P(X_{t+1}\leq x|I_{t})=P(X_{t+1}\leq x|X_{t})\;\;\mathrm{almost}\;\mathrm{surely}\;\mathrm{for}\;\mathrm{all}\;x\in R^{d}\;\mathrm{and}\;t>0;\\ & \;H_{A}\;:\;P(X_{t+1}\leq x|I_{t})\neq P(X_{t+1}\leq x|X_{t})\;\;\mathrm{for}\;\mathrm{some}\;x\in R^{d}\;\mathrm{and}\;t>0, \end{array}$$

where $I_{t}$ denotes the data history $\left\{X_{t},X_{t-1},\ldots \right\}$. The Markov property holds under $H_{0}$. Intuitively, this property requires the past and future values to be independent, conditionally on the present. To test $H_{0}$, it suffices to test a sequence of conditional independences


(2) $$X_{t+q}⊥\{X_{\;j}{\}}_{t\leq j<t+q-1}\;|\;X_{t+q-1},$$

for any time t>0 and any lag q≥2, where ⊥ denotes the conditional independence.

We next characterise the conditional independence using the CCF. A similar result is given in Chen and Hong (2012, Equation (2.6)). For any vector $\mu \in R^{d}$ of the same dimension as $X_{t}$, define the CCF of $X_{t+1}$ given $X_{t}$ as


$$\varphi^{*}(\mu |x)=E\left\{\exp\;(i\mu^{⊤}X_{t+1})|X_{t}=x\right\}.$$

Theorem 1 The conditional independence (2) holds if and only if

(3) $$\begin{array}{r} \varphi^{*}(\mu |X_{t+q-1})E[\exp\;(i\nu^{⊤}X_{t})|\{X_{j}{\}}_{t<j<t+q}]=E\left[\exp\;(i\mu^{⊤}X_{t+q}+i\nu^{⊤}X_{t})|\{X_{j}{\}}_{t<j<t+q}\right] \end{array}$$

almost surely, for any t>0, q≥2, and $\mu ,\nu \in R^{d}$.

### 2.2 Doubly robust test statistic

Theorem 1 suggests a possible test for the hypotheses in (1). That is, under $H_{0}$, taking another expectation on both sides of (3), we obtain that


$$E\left[\left\{\exp\;(i\mu^{⊤}X_{t+q})-\varphi^{*}(\mu |X_{t+q-1})\right\}\exp\;(i\nu^{⊤}X_{t})\right]=0,$$

for any t,q,μ,ν. This suggests the following test statistic:


(4) $$\tilde{S}(q,\mu ,\nu )=\frac{1}{T-q}\sum_{t=1}^{T-q}\left\{\exp\;(i\mu^{⊤}X_{t+q})-\hat{\varphi }(\mu |X_{t+q-1})\right\}\left\{\exp\;(i\nu^{⊤}X_{t})-\bar{\varphi }(\nu )\right\},$$

where $\hat{\varphi }$ denotes some estimator of the CCF $\varphi^{*}$, and $\bar{\varphi }(\nu )=T^{-1}\sum_{1\leq t\leq T}\exp\;(i\nu^{⊤}X_{t})$. Aggregating $\tilde{S}(q,\mu ,\nu )$ over different combinations of (q,μ,ν) yields the test statistic proposed in Chen and Hong (2012, Equation (2.18)).

Computing (4) requires a suitable estimator $\hat{\varphi }$ for $\varphi^{*}$. Chen and Hong (2012) proposed to use the LPR to estimate $\varphi^{*}$. However, the LPR tends to perform poorly when the dimension *d* of $X_{t}$ increases (Taylor & Einbeck, 2013), and the corresponding test would fail to be consistent. More recently, deep conditional generative learning models have demonstrated an exceptional capacity of estimating complex conditional distributions (e.g. Kobyzev et al., 2020; Sohn et al., 2015). These tools can be potentially employed to estimate $P_{X_{t}|X_{t-1}}$, and subsequently the CCF $\varphi^{*}$. However, naively plugging in a deep conditional generative learning estimator for $\varphi^{*}$ would induce a heavy bias in (4), which would fail to guarantee a tractable limiting distribution for the test statistic.

To address this issue, we propose to construct a doubly robust test statistic. Specifically, for any vector $\nu \in R^{d}$ of the same dimension as $X_{t}$, define the CCF of $X_{t}$ given $X_{t+1}$ as


$$\psi^{*}(\nu |x)=E\left\{\exp\;(i\nu^{⊤}X_{t})|X_{t+1}=x\right\}.$$

We introduce a doubly robust estimating equation in the next theorem.

Theorem 2 Under $H_{0}$, for any t≥0, q≥2, $\mu ,\nu \in R^{d}$, we have

(5) $$E\left\{\exp\;(i\mu^{⊤}X_{t+q})-\varphi^{*}(\mu |X_{t+q-1})\right\}\left\{\exp\;(i\nu^{⊤}X_{t})-\psi^{*}(\nu |X_{t+1})\right\}=0.$$

In addition, (5) is doubly robust, in that, for any CCFs *φ* and *ψ*, as long as either $\varphi =\varphi^{*}$, or $\psi =\psi^{*}$, we have that $E\{\;\exp\;(i\mu^{⊤}X_{t+q})-\varphi (\mu |X_{t+q-1})\}\{\;\exp\;(i\nu^{⊤}X_{t})-\psi (\nu |X_{t+1})\}=0$.

Motivated by (5), we propose the following test statistic:


(6) $$S(q,\mu ,\nu )=\frac{1}{T-q}\sum_{t=1}^{T-q}\{\;\exp\;(i\mu^{⊤}X_{t+q})-\hat{\varphi }(\mu |X_{t+q-1})\}\{\exp\;(i\nu^{⊤}X_{t})-\hat{\psi }(\nu |X_{t+1})\},$$

where $\hat{\varphi }$ and $\hat{\psi }$ denote some estimators of $\varphi^{*}$ and $\psi^{*}$, respectively. This statistic, as suggested by Theorem 2, is doubly robust. A key advantage is that the bias of this test statistic can decay to zero at a faster rate than the convergence rate of the individual estimator $\hat{\varphi }$ and $\hat{\psi }$. By contrast, the bias of the test statistic in (4) has the same order of magnitude as that of $\hat{\varphi }$; see Theorem 4. This double robustness property thus enables us to employ some highly flexible nonparametric estimators for $\varphi^{*}$ and $\psi^{*}$. In the next section, we extend MDNs (Bishop, 1994) to estimate the CCFs, and develop the corresponding testing procedure.

## 3 Testing procedure

### 3.1 Mixture density networks

The MDN is a classical deep generative model that combines the Gaussian mixture model with deep neural networks (DNNs) (Bishop, 1994), and has shown promising performance in conditional density estimation (Koohababni et al., 2018; Rothfuss et al., 2019). In effect it integrates the universal approximation property of the Gaussian mixture model to approximate any smooth density function (Nguyen & McLachlan, 2019), with the capacity of DNNs to approximate both smooth and nonsmooth conditional mean and variance functions in high dimension. See Assumption 2(iii) for the class of smooth functions, and Imaizumi and Fukumizu (2019) for the class of nonsmooth functions that can be well approximated by DNNs. Next, we first introduce the standard MDN model, then propose a new MDN architecture to model the conditional distribution of a multivariate response.

**Figure 1.**
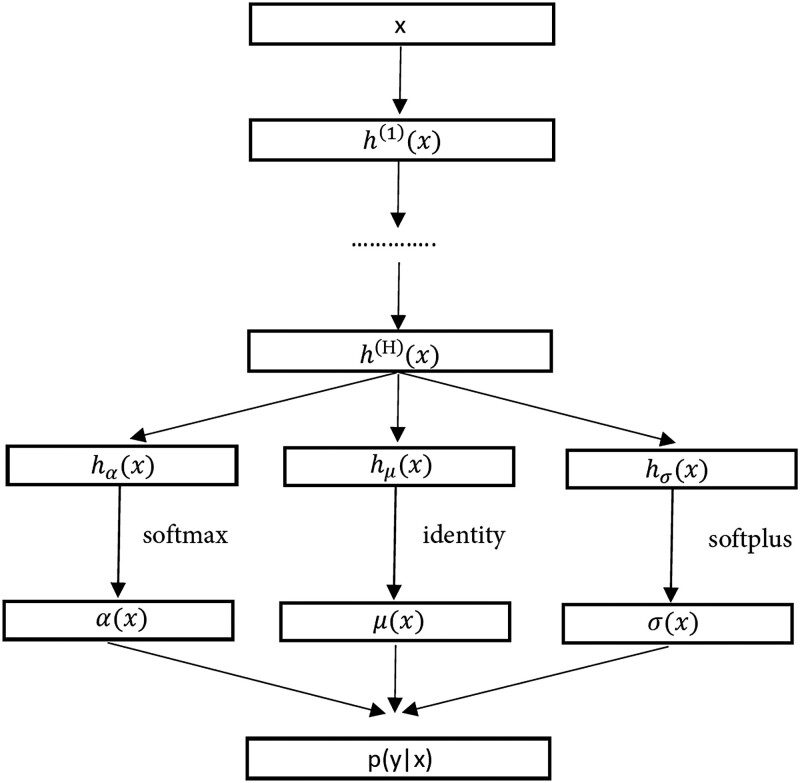
Structure of the MDN.

We aim to estimate an unknown conditional probability density function of some univariate response *Y* given a predictor vector $X\in R^{d_{0}}$ with $d_{0}$ being the input dimension. Suppose the conditional density of *Y* given *X* follows a MDN model,


(7) $$f(y|x)=\sum_{g=1}^{G}\alpha_{g}(x)\frac{1}{\sqrt{2\pi }\sigma_{g}(x)}\exp\;\left[-\frac{\{y-\mu_{g}(x){\}}^{2}}{2\sigma_{g}^{2}(x)}\right],$$

where *G* is the number of mixture components, and DNNs are used to estimate the mean vector $\mu (x)=(\mu_{1}(x),\ldots ,\mu_{G}(x){)}^{⊤}$, the standard deviation vector $\sigma =(\sigma_{1}(x),\ldots ,\sigma_{G}(x){)}^{⊤}$, and the weight vector $\alpha =(\alpha_{1}(x),\ldots ,\alpha_{G}(x){)}^{⊤}$. Figure 1 depicts the structure of the model. The input layer is the $d_{0}$-dimension vector *x*. Then, there are *H* hidden layers, each with a number of hidden units. A hidden layer is between the input and output layers, which takes in a set of weighted inputs and produces an output through an activation function. The last hidden layer outputs a *G*-dimensional vector $h^{\left(H\right)}(x)$, and is connected to three parallel layers whose outputs are given by


$$h_{\alpha }(x)=\Theta_{1}^{⊤}h^{\left(H\right)}(x),\;h_{\mu }(x)=\Theta_{2}h^{\left(H\right)}(x),\;h_{\sigma }(x)=\Theta_{3}^{⊤}h^{\left(H\right)}(x),$$

respectively, where $\Theta_{j}$ is a G×G coefficient matrix that is to be trained via back propagation, j=1,2,3. Next, two of those functions pass through activation functions, yielding


$$\alpha (x)=\text{softmax}(h_{\alpha }(x)),\;\mu (x)=h_{\mu }(x),\;\sigma (x)=\text{softplus}(h_{\sigma }(x)),$$

respectively, where α(x), $h_{\alpha }(x)$, μ(x), $h_{\mu }(x)$, σ(x), and $h_{\sigma }(x)$ are all *G*-dimensional vectors, and the activation functions are applied in an element-wise fashion. Finally, all these components are combined to parametrise f(y|x) according to (7) with a total of *W* parameters.

Next, we propose a new MDN architecture to model the conditional density of a multivariate response variable $Y\in R^{d_{y}}$. The main idea is to factorise the joint conditional density function f(y|x) as the product of $d_{y}$ conditional densities, each with a univariate response,


(8) $$f(y|x)=f_{1}(y_{1}|x)f_{2}(y_{2}|x,y_{1})\cdots f_{d_{y}}(y_{d_{y}}|x,y_{1},y_{2},\ldots ,y_{d_{y}-1}).$$

It then suffices to model each $f_{j}(y_{j}|x,y_{1},\ldots ,y_{\;j-1})$ separately. When the individual component of *Y* is a continuous variable, we use the MDN model (7) to estimate the conditional density, whereas when it is a categorical variable, we use a supervised learning method, such as a random forest (Breiman, 2001), or a DNN (LeCun et al., 2015) to estimate the probability mass function. We briefly note that, Bishop (1994) also considered a version of MDN for the multivariate response, by extending (7) to a mixture of multivariate normal densities. However, such an extension does not work well when the components of the response have mixed type of continuous and categorical variables.

We also comment that, most of the existing MDN literature study i.i.d. data. In our setting, the observed data are time-dependent. We later show that MDN is equally applicable, as long as the time series satisfies some mixing conditions such as *β*-mixing (Wu & Shao, 2004).

### 3.2 Testing Markov property

Next, we develop a testing procedure for the hypotheses in (1), where the key idea is to build upon the doubly robust test statistic (6) and estimate the CCFs using MDN. Moreover, to avoid requiring the estimators of the CCFs to satisfy some restrictive metric entropy conditions, we employ the sample splitting and cross-fitting strategy. We first summarise our testing procedure in Algorithm 1, then discuss the main steps in detail.

In Step 1 of the algorithm, we divide the time series into *L* nonoverlapping chunks of similar sizes. For simplicity, suppose the length *T* of the observed time series is a multiple of *L*, and let n=T/L. Let $I^{\left(\ell \right)}=\{(\ell -1)n+1,(\ell -1)n+2,\ldots ,\ell n\}$ denote the indices of the ℓth chunk of the time series, and let ${\bar{I}}^{\left(\ell \right)}=\cup_{\;j=1}^{\ell }I^{\left(j\right)}$ denote the union of indices of the first ℓ chunks, ℓ=1,…,L. Data splitting allows us to use part of the data, i.e. the data ${\bar{I}}^{\left(\ell \right)}$ up to chunk ℓ, to train the MDN model, and another part, i.e. $I^{\left(\ell +1\right)}$, to construct the test statistic. We then aggregate the estimates over all chunks to improve the estimation efficiency.

**Algorithm 1:** Testing procedure for the Markov property

**Input**: Data $\{X_{t}{\}}_{t=1,\ldots ,T}$, the number of data chunks *L*, the number of pairs *B*, the largest number of lags *Q*, and the number of samples from the generators *M*.
**Step 1**: Divide the time series data into *L* nonoverlapping chunks, where n=T/L, $I^{\left(\ell \right)}=\{(\ell -1)n+1,(\ell -1)n+2,\ldots ,\ell n\}$, and ${\bar{I}}^{\left(\ell \right)}=\cup_{\;j=1}^{\ell }I^{\left(j\right)}$, ℓ=1,…,L.
**Step 2**: Deep conditional forward–backward generative learning.
(2a) Obtain the estimators of a forward generator ${\hat{\;f}}_{X_{t}|X_{t-1}}^{\left(\ell \right)}$, and a backward generator ${\hat{\;f}}_{X_{t-1}|X_{t}}^{\left(\ell \right)}$, using the data ${\bar{I}}^{\left(\ell \right)}$ up to chunk ℓ, ℓ=1,…,L−1.
(2b) Randomly sample *M* copies of *d*-dimensional time series observations $\{X_{m,f}^{*}{\}}_{m=1}^{M}$ and $\{X_{m,b}^{*}{\}}_{m=1}^{M}$ from each generator.
(2c) Randomly sample *B* pairs $\{(\mu_{b},\nu_{b}){\}}_{1\leq b\leq B}$ from a multivariate normal distributions with zero mean and identity covariance matrix.
(2d) Compute the CCF estimators ${\hat{\varphi }}^{\left(\ell \right)}(\mu_{b}|x)$ and ${\hat{\psi }}^{\left(\ell \right)}(\nu_{b}|x)$ according to (9), for ℓ=1,…,L−1, and b=1,…,B.
**Step 3**: Construct the test statistic.
(3a) Compute $\hat{S}(q,\mu_{b},\nu_{b})$ according to (10), for q=2,…,Q, b=1,…,B.
(3b) Construct the test statistic $\hat{S}$ according to (11).
**Step 4**: Compute the critical value.
(4a) Compute the covariance matrix ${\hat{\Sigma }}^{\left(q\right)}$ according to (12), for q=2,…,Q.
(4b) Compute the critical value according to (13).
**Step 5**: Reject $H_{0}$ if $\hat{S}$ is greater than ${\hat{c}}_{\alpha }$.

In Step 2, we employ MDN to estimate the CCFs. Specifically, for each subset ℓ=1,…,L−1, we first apply MDN to the data ${\bar{I}}^{\left(\ell \right)}$ up to the ℓth chunk to obtain the estimates of two conditional probability density functions, a forward generator ${\hat{\;f}}_{X_{t}|X_{t-1}}^{\left(\ell \right)}$, and a backward generator ${\hat{\;f}}_{X_{t-1}|X_{t}}^{\left(\ell \right)}$. For the forward generator, the ‘predictor’ for the MDN model (8) is $(X_{1},X_{2},\ldots ,X_{\ell n-1}{)}^{⊤}$ and the ‘response’ is $(X_{2},X_{3},\ldots ,X_{\ell n}{)}^{⊤}$, whereas for the backward generator, the ‘predictor’ for (8) is $(X_{2},X_{3},\ldots ,X_{\ell n}{)}^{⊤}$ and the ‘response’ is $(X_{1},X_{2},\ldots ,$  $X_{\ell n-1}{)}^{⊤}$. Given the two estimated density functions ${\hat{\;f}}_{X_{t}|X_{t-1}}^{\left(\ell \right)}$ and ${\hat{\;f}}_{X_{t-1}|X_{t}}^{\left(\ell \right)}$, we then randomly sample *M* copies of *d*-dimensional time series observations $\{X_{m,f}^{*}{\}}_{m=1}^{M}$ and $\{X_{m,b}^{*}{\}}_{m=1}^{M}$, respectively. Next, we consider different combinations of (μ,ν) for the test statistic S(q,μ,ν) in (6). Toward that end, we randomly sample *B* i.i.d. pairs of $\{(\mu_{b},\nu_{b}){\}}_{b=1}^{B}$ from a multivariate normal distribution with zero mean and identity covariance matrix. Finally, by noting that $\varphi^{*}(\mu |x)=E\{\;\exp\;(i\mu^{⊤}X_{t})|X_{t-1}=x\}$ and $\psi^{*}(\nu |x)\;=E\{\;\exp\;(i\mu^{⊤}X_{t-1})|X_{t}=x\}$, we obtain the Monte Carlo estimators of $\varphi^{*}(\mu |x)$ and $\psi^{*}(\nu |x)$ for each pair of $\left(\mu_{b},\nu_{b}\right)$ as


(9) $${\hat{\varphi }}^{\left(\ell \right)}(\mu_{b}|x)=\frac{1}{M}\sum_{m=1}^{M}\exp\;(i\mu_{b}^{⊤}X_{m,f}^{*}),\;{\hat{\psi }}^{\left(\ell \right)}(\nu_{b}|x)=\frac{1}{M}\sum_{m=1}^{M}\exp\;(i\nu_{b}^{⊤}X_{m,b}^{*}).$$

Due to the use of both forward and backward generators and DNNs, we refer to this step as deep conditional forward–backward generative learning.

In Step 3, we construct our final composite test statistic given the estimates of ${\hat{\varphi }}^{\left(\ell \right)}(\mu_{b}|x)$ and ${\hat{\psi }}^{\left(\ell \right)}(\nu_{b}|x)$. We first compute S(q,μ,ν) in (6) using the cross-fitting strategy, i.e.


(10) $$\begin{array}{rl} \hat{S}(q,\mu_{b},\nu_{b}) & =\frac{1}{T-n-(q-1)(L-1)}\sum_{\ell =1}^{L-1}\;\sum_{t=1}^{n-q+1}\left\{\exp\;(i\mu_{b}^{⊤}X_{\ell n+t+q-1}\right)\\ & \;-\;{\hat{\varphi }}^{\left(\ell \right)}(\mu_{b}|X_{\ell n+t+q-2})\}\left\{\exp\;(i\nu_{b}^{⊤}X_{\ell n+t-1})-{\hat{\psi }}^{\left(\ell \right)}(\nu_{b}|X_{\ell n+t})\right\}, \end{array}$$

for a given q=2,…,Q, and *Q* denotes the largest number of lags to consider in the test. We note that, for any given ℓ=1,…,L−1, the set of random variables $\{X_{\ell n+t}{\}}_{1\leq t\leq n}$ that appear in (10) are from the (ℓ+1)th chunk of the data, and are, under $H_{0}$, independent of ${\hat{\varphi }}^{\left(\ell \right)}$ and ${\hat{\psi }}^{\left(\ell \right)}$ given $X_{\ell n+1}$. This allows us to avoid imposing certain entropy growth condition that limits the growth rate of the VC dimension of the MDN model with respect to the sample size (Chernozhukov et al., 2018). A similar cross-fitting procedure has also been utilised by Luedtke and Van Der Laan (2016) and Shi et al. (2022) for evaluation of an optimal policy, as well as by Luedtke and Van Der Laan (2018) and Shi et al. (2021) for high-dimensional statistical inference. Next, since $\hat{S}(q,\mu_{b},\nu_{b})$ is complex-valued, we use ${\hat{S}}_{R}(q,\mu_{b},\nu_{b})$ and ${\hat{S}}_{I}(q,\mu_{b},\nu_{b})$ to denote its real and imaginary part, respectively. We construct our final test statistic as


(11) $$\hat{S}=\max_{b\in \{1,\ldots ,B\}}\max_{q\in \{2,\ldots ,Q\}}\sqrt{T-n-(q-1)(L-1)}\max\left(|{\hat{S}}_{R}(q,\mu_{b},\nu_{b})|,|{\hat{S}}_{I}(q,\mu_{b},\nu_{b})|\right).$$

In (11), we take the maximum absolute value over multiple combinations of $\left(q,\mu_{b},\nu_{b}\right)$ to construct the test statistic, while we generate $\mu_{b}$ and $\nu_{b}$ from a Gaussian or uniform distribution. This way, we do not have to impose a bounded support for $\left(\mu_{b},\nu_{b}\right)$, and avoid grid search that can be computationally intensive in a high-dimensional setting.

In Step 4, we compute the critical value of the test statistic $\hat{S}$. A key observation is that, under $H_{0}$, each ${\hat{S}}_{R}(q,\mu_{b},\nu_{b})$ and ${\hat{S}}_{I}(q,\mu_{b},\nu_{b})$ corresponds to a sum of martingale difference sequences. Since the sum of martingale difference is a martingale (Hamilton, 2020), it follows from the high-dimensional martingale central limit theorem that $\hat{S}$ converges in distribution to a maximum of some Gaussian random variables. This allows us to employ the high-dimensional multiplier bootstrap method of Belloni and Oliveira (2018) to estimate the critical value. Specifically, we stack ${\hat{S}}_{R}(q,\mu_{b},\nu_{b})$ and ${\hat{S}}_{I}(q,\mu_{b},\nu_{b})$ for a given *q* and all b=1,…,B together to form a 2B-dimensional vector, and estimate the covariance matrix of this vector by


(12) $${\hat{\Sigma }}^{\left(q\right)}=\sum_{\ell =1}^{L-1}\sum_{t=1}^{n-q+1}\frac{\left(\lambda_{R,\ell ,q,t}^{⊤},\lambda_{I,\ell ,q,t}^{⊤}{)}^{⊤}(\lambda_{R,\ell ,q,t}^{⊤},\lambda_{I,\ell ,q,t}^{⊤}\right)}{\left(T-n-(q-1)(L-1)\right)},$$

where $\lambda_{R,\ell ,q,t},\lambda_{I,\ell ,q,t}$, ℓ=1,…,L−1, t=1,…,n−q+1, are both *B*-dimensional vectors, whose *b*th element is, respectively, the real and imaginary part of


$$\left\{\exp\;(i\mu^{⊤}X_{t+q-1+\ell n})-{\hat{\varphi }}^{\left(-\ell \right)}(\mu |X_{t+q-2+\ell n})\right\}\left\{\exp\;(i\nu^{⊤}X_{t-1+\ell n})-{\hat{\psi }}^{\left(-\ell \right)}(\nu |X_{t+\ell n})\right\}.$$

We then compute the critical value ${\hat{c}}_{\alpha }$ by simulating the upper (α/2)th critical value of


(13) $$\max_{q\in \{2,\ldots ,Q\}}{\left\|\{{\hat{\Sigma }}^{\left(q\right)}{\}}^{1/2}Z_{q}\right\|}_{\infty },$$

using Monte Carlo, where $Z_{0},\ldots ,Z_{Q}$ are i.i.d. 2B-dimensional standard normal vectors.

In Step 5, we reject $H_{0}$, if $\hat{S}>{\hat{c}}_{\alpha }$, under a given significance level α>0.

We make a few remarks. First, in terms of the computational cost, step 2(a) is the most intensive step in Algorithm 1, as it involves fitting multiple MDN models. Second, there are a number of hyper-parameters in our test, including the number of mixture components *G*, the number of data chunks *L*, the number of pairs *B* of (μ,ν), the number of samples *M* from the forward and backward generators, and the largest number of lags *Q* considered in the test. We proposed to choose *G* using cross-validation, and take the rest as the input parameters. We further discuss their theoretical choices in Section 4, and their empirical choices in Section 5.

### 3.3 Determining Markov order

The proposed test can be used to determine the order of the Markov model. Specifically, let $X_{t}^{\left(k\right)}=(X_{t}^{⊤},\ldots ,X_{t+k-1}^{⊤}{)}^{⊤}$ denote the multivariate time series that concatenates the most recent *k* observations at each time point. Suppose the data follows a *K*th order Markov model. Then the null hypothesis $H_{0}$ holds for the concatenated time series $X_{t}^{\left(k\right)}$ for any k≥K, but does not hold for any k<K. This suggests we can sequentially test the Markov property on the concatenated time series $X_{t}^{\left(k\right)}$ for k=1,2,…. We set the estimated order to be the first integer *k* by which we fail to reject $H_{0}$. We also briefly remark that *K* is different from *Q*. The former denotes the largest possible order of the underlying Markov model, whereas the latter denotes the largest number of lags considered in our test for a series of conditional dependences.

## 4 Theory

### 4.1 Convergence rate of MDN

We first establish the error bound of the MDN estimator, then establish the consistency of the proposed test. We begin with some regularity conditions, and argue they are relatively mild and reasonable.

Let $f_{X_{t+1}|X_{t}}^{*}(\cdot |x)$ and $f_{X_{t}|X_{t+1}}^{*}(\cdot |x)$ denote the true conditional density function of $X_{t+1}$ given $X_{t}=x$, and that of $X_{t}$ given $X_{t+1}=x$, respectively. A key observation is that $f_{X_{t+1}|X_{t}}^{*}={\arg\;\max}_{f}E[\log\;\{f(X_{t+1}|X_{t})\}]$, and $f_{X_{t}|X_{t+1}}^{*}={\arg\;\max}_{f}E[\log\;\{f(X_{t}|X_{t+1})\}]$, where *f* belongs to a Sobolev ball with the smoothness $\gamma \in N_{+}\;:\;\{f\;:\;\max_{\nu ,\|\nu {\|}_{1}\leq \gamma }\underset{\begin{array}{c} x \end{array}}{\sup}|D^{\nu }f(x)|<+\infty \}$. Given the data ${\bar{I}}^{\left(\ell \right)}$ up to chunk ℓ, the estimated density functions are


$${\hat{\;f}}_{X_{t+1}|X_{t}}^{\left(\ell \right)}=\underset{f}{\arg\;\max}\;\sum_{t=1}^{T-1}\log\;\{f(X_{t+1}|X_{t})\},\;{\hat{\;f}}_{X_{t}|X_{t+1}}^{\left(\ell \right)}=\underset{f}{\arg\;\max}\;\sum_{t=1}^{T-1}\log\;\{f(X_{t}|X_{t+1})\},$$

based on the maximum likelihood. In the following, we focus on establishing the statistical properties of ${\hat{\;f}}_{X_{t+1}|X_{t}}^{\left(\ell \right)}$. The properties of ${\hat{\;f}}_{X_{t}|X_{t+1}}^{\left(\ell \right)}$ can be derived in similar manner.

Assumption 1 Suppose the following conditions hold for the time series $X_{t}$.

- Let $X_{t}$ be stationary, and its *β*-mixing coefficient satisfy the that $\beta (t)\leq c_{1}\exp\;(\;-\;c_{2}t)$ for some constants $c_{1},c_{2}>0$.
- Let X denote the support of $X_{t}$, and X be a compact subset of $R^{d}$.

Assumption 1(i) requires the *β*-mixing coefficient to decay exponentially with respect to *t*. Under the Markov property, it is equivalent to the geometric ergodicity condition (Bradley, 2005). Such a condition is commonly imposed in the time series literature (see, e.g. Cline & Pu, 1999; Liebscher, 2005; Wu & Shao, 2004). We also note that the *β*-mixing condition is not limited to a Markov process. For instance, Neumann (2011) considered a class of observation-driven Poisson count process, which is *β*-mixing but non-Markovian.

Assumption 2 Suppose the following conditions hold for the true density function $f_{X_{t+1}|X_{t}}^{*}$.

- Suppose $f_{X_{t+1}|X_{t}}^{*}(y|x)$ can be well approximated by a conditional Gaussian mixture model with *G* components, in that, there exists some constant $\omega_{1}>0$, such that
  $$\left|f_{X_{t+1}|X_{t}}^{*}(y|x)-\sum_{g=1}^{G}\frac{\alpha_{g}^{*}(x)}{\sqrt{2\pi }\sigma_{g}^{*}(x)}\exp\;\left\{-\frac{(y-\mu_{g}^{*}(x){)}^{2}}{2{\sigma_{g}^{*}}^{2}(x)}\right\}\right|=O(G^{-\omega_{1}}),$$
  where the big-*O* term is uniform in *x* and *y*.
- Suppose $\{\mu_{g}^{*}{\}}_{g=1}^{G}$ and $\{\sigma_{g}^{*}{\}}_{g=1}^{G}$ are uniformly bounded away from infinity, and there exist a constant $C_{0}>0,\omega_{2}\geq 0$, such that $\sigma_{g}^{*}(x)\geq C_{0}G^{-\omega_{2}}$ for any *g* and *x*.
- Suppose $\alpha_{g}^{*}(\cdot )$, $\mu_{g}^{*}(\cdot )$, and $\sigma_{g}^{*}(\cdot )$, g=1,…,G, all lie in the Sobolev ball with the smoothness $\gamma \in N_{+}\;:\;\{f\;:\;\max_{\nu ,\|\nu {\|}_{1}\leq \gamma }\underset{\begin{array}{c} x \end{array}}{\sup}|D^{\nu }f(x)|<+\infty \}$, where the maximum is taken over all *d*-dimensional non-negative integer-valued vectors ν the sum of whose elements is no greater than γ, and $D^{\alpha }f$ is the weak derivative (Giné & Nickl, 2015).
- Suppose $f_{X_{t+1}|X_{t}}^{*}(\cdot |\cdot )$ is uniformly bounded away from zero on X×X.

Assumption 2(i) requires the true conditional density function $f_{X_{t+1}|X_{t}}^{*}$ can be well approximated by a conditional Gaussian mixture model, with a sufficiently large number of components *G*. This is reasonable, since the Gaussian mixture model can approximate any smooth density function, and the conditional Gaussian mixture model can approximate any smooth conditional density function (Dalal & Hall, 1983). Assumption 2(ii) to (iv) impose certain boundedness and smoothness conditions on the mean, variance, and weight functions used in the approximation of $f_{X_{t+1}|X_{t}}^{*}$, as well as on $f_{X_{t+1}|X_{t}}^{*}$ itself. All these conditions are reasonably mild and hold under numerous settings. We consider three examples to further illustrate.

Example 1 Suppose the true conditional density function $f_{X_{t+1}|X_{t}}^{*}$ follows a finite conditional Gaussian mixture model with bounded and smooth mean, variance, and weight functions. Then Assumption 2 trivially holds.

Example 2 Suppose $f_{X_{t+1}|X_{t}}^{*}$ follows an infinite conditional Gaussian mixture model, i.e.

(14) $$f_{X_{t+1}|X_{t}}^{*}(y|x)=\int g(y_{0}|x)\phi_{\sigma }(y-y_{0})dy_{0},$$

where *g* denotes a certain conditional density function, and $\phi_{\sigma }$ denotes the probability density function of a Gaussian random variable with mean zero and variance $\sigma^{2}$. Then under some mild conditions on *g*, the next lemma show that Assumption 2 holds.

Lemma 1 Suppose (14) holds, with a conditional density function *g* bounded away from infinity. Suppose the support of g(⋅|x) is a subset of $\left[-C_{1},C_{1}\right]$ for any *x*. It follows that

$$\left|f_{X_{t+1}|X_{t}}^{*}(y|x)-\sum_{g=1}^{G}\alpha_{g}^{*}(x)\phi_{\sigma }\left(y+C_{1}-\frac{2C_{1}(g-1)}{G}\right)\right|\leq c_{4}G^{-1},$$

where $\alpha_{g}^{*}(x)=\int_{-C_{1}+\frac{2C_{1}(g-1)}{G}}^{-C_{1}+\frac{2C_{1}g}{G}}\;g(z|x)dz$, and $c_{4}$ is a positive constant independent of *x* and *y*.

According to Lemma 1, the mean $\{\mu_{g}^{*}(x){\}}_{g=1}^{G}$ and variance $\{\sigma_{g}^{*}(x){\}}_{g=1}^{G}$ are constant functions of *x*, which are equal to $2C_{1}(g-1)/K-C_{1}$ and *σ*. Then Assumption 2(i) holds with $\omega_{1}=1$, and Assumption 2(ii) holds with $\omega_{2}=0$. When *g* lies in the Sobolev ball with the smoothness parameter *γ*, so are the weight functions $\{\alpha_{g}^{*}{\}}_{g=1}^{G}$, and Assumption 2(iii) holds. Assumption 2(iv) holds as *g* is bounded away from zero. Besides, the approximation error rate obtained in Lemma 1 is $O(G^{-1})$ in $L_{\infty }$ norm, which is shaper than the $O(G^{-1/2})$ rate in $L_{2}$ norm obtained in Barron (1993, Lemma 1), as we focus on the Gaussian mixture and one-dimensional case.

Example 3 Suppose $f_{X_{t+1}|X_{t}}^{*}$ satisfies Assumption 2(iv), and is Lipschitz continuous, i.e. $\left|f^{*}(y_{1}|x)-f^{*}(y_{2}|x)|=O(|y_{1}-y_{2}|\right)$ where the big-*O*-term is uniform in *x*. It follows from Nguyen and McLachlan (2019, Theorem 9) that $f^{*}$ can be well approximated by an infinite conditional Gaussian mixture model specified in (14) with $g=f^{*}$, with the approximation error O(σ). In addition, similar to Lemma 1, we can show that this infinite conditional Gaussian mixture model can be approximated by the finite conditional Gaussian mixture model, with the approximation error $O(\sigma^{-1}G^{-1})$. By setting $\sigma =G^{-1/2}$, Assumption 2(i) holds with $\omega_{1}=1/2$. The mean and variance are both constant functions of *x*, and the variance is lower bounded by $G^{-1/2}$. Assumption 2(ii) thus holds with $\omega_{2}=1/2$. When $f^{*}$ lies in the Sobolev ball with the smoothness parameter *γ*, so are the weight functions $\alpha_{g}^{*}$, and Assumption 2(iii) holds.

Assumption 3 Suppose the following conditions hold for the MDN model.

- Suppose the MDN function class is given by, for some sufficiently large constant $C_{2}$,
  F={f(y|x)=∑g=1Gαg(x)2πσg(x)exp{−(y−μg(x))22σg2(x)}:infx,yf(y|x)≥C2−1,∑g=1Gsupx,g|μg(x)|≤C2,C2−1G−ω2≤infx,gσg(x)≤supx,gσg(x)≤C2},
  where $\alpha_{g}$, $\mu_{g}$ and $\sigma_{g}$ are parametrised via DNNs.
- The total number of parameters *W* in the MDN model is proportional to $G^{(d+\gamma )/\gamma }T^{d/(2\gamma +d)}$  log(GT), where *γ* is the smoothness parameter specified in Assumption 2(iii).

Assumption 3(i) is mainly to simplify the technical proof, since the estimated functions are bounded when both the model parameters and the data support are bounded. It is easy to enforce Assumption 3(i) in practice, by imposing range constraints on the model parameters. Assumption 3(ii) specifies the total number of parameters *W*, which represents a trade-off. On one hand, since we model $\{\alpha_{g}{\}}_{g=1}^{G}$, $\{\mu_{g}{\}}_{g=1}^{G}$ and $\{\sigma_{g}{\}}_{g=1}^{G}$ via DNNs, their approximation errors decay as *W* increases. On the other hand, the estimation error of MDN increases with *W*. We require *W* to be proportional to $G^{(d+\gamma )/\gamma }T^{d/(2\gamma +d)}\log\;(GT)$ to balance the bias-variance trade-off, and optimise the convergence rate of the MDN estimator. See the proof of Theorem 3 in the Online Supplementary Material, Appendix, for more details.

Next, we establish the error bound of the MDN estimator ${\hat{\;f}}_{X_{t+1}|X_{t}}^{\left(\ell \right)}$. The bound of ${\hat{\;f}}_{X_{t}|X_{t+1}}^{\left(\ell \right)}$ is the same and can be derived similarly.

Theorem 3 Suppose Assumptions 1 and 2 hold. Then, there exist a certain MDN function class satisfying Assumption 3, such that the resulting MDN estimator ${\hat{\;f}}_{X_{t+1}|X_{t}}^{\left(\ell \right)}$ satisfies that

(15) $$\begin{array}{rl} {\left\|{\hat{\;f}}_{X_{t+1}|X_{t}}^{\left(\ell \right)}-f_{X_{t+1}|X_{t}}^{*}\right\|}_{2} & =\sqrt{\int_{x,y}|{\hat{\;f}}_{X_{t+1}|X_{t}}^{\left(\ell \right)}(y|x)-f_{X_{t+1}|X_{t}}^{*}(y|x){|}^{2}dxdy}\\ & \leq cd\left\{G^{-\omega_{1}}+G^{\frac{\gamma +d}{2\gamma }+4\omega_{2}}T^{-\frac{\gamma }{2\gamma +d}}\log^{3}\;(TG)\right\}, \end{array}$$

for some constant c>0, and any ℓ=1,…,L, with probability at least $1-O(T^{-1})$.

We remark that the first term of the error bound in (15) is due to the approximation error of the conditional Gaussian mixture model, while the second term is due to the approximation error of the DNNs and the estimation error of the MDN estimator. In general, the error bound increases with *d* and $\omega_{2}$, and decreases with *γ* and $\omega_{1}$. We next revisit Examples 1 to 3, and discuss the corresponding rate of convergence.

**Example 1 revisited.** In this example, the finite conditional Gaussian mixture model holds. As a result, *G* is finite and $\omega_{1}$ can be chosen arbitrarily large. The error bound is then of the same order of magnitude as $dT^{-\gamma /\{2\gamma +d\}}\log^{3}\;(T)$. If the mean, variance, and weight functions are infinitely differentiable, i.e. γ=+∞, then the MDN estimator achieves a convergence rate of $dT^{-1/2}$ up to some logarithmic term.

**Example 2 revisited.** In this example, the infinite conditional Gaussian mixture model holds. As a result, $\omega_{1}=1$ and $\omega_{2}=0$. By setting *G* to be proportional to $T^{2\gamma^{2}/\{(2\gamma +d)(3\gamma +d)\}}$, the error bound is minimised and is proportional to $dT^{-2\gamma^{2}/\{(2\gamma +d)(3\gamma +d)\}}\log^{3}\;(T)$. If γ=+∞, then the MDN estimator achieves a convergence rate of $dT^{-1/3}$ up to some logarithmic term.

**Example 3 revisited.** In this example, we have $\omega_{1}=\omega_{2}=1/2$. The error bound is minimised when *G* is proportional to $T^{2\gamma^{2}/\{(2\gamma +d)(6\gamma +d)\}}$, and the resulting convergence rate is $dT^{-\gamma^{2}/\{(2\gamma +d)(6\gamma +d)\}}\log^{3}\;(T)$. If γ=+∞, then the MDN estimator achieves a convergence rate of $dT^{-1/12}$ up to some logarithmic term.

Finally, we remark on the problem of determining the order of a Markov model. In this case, we are interested in estimating the conditional density function of $X_{t+K}$ given $X_{t}^{\left(K\right)}$ and $X_{t-1}$ given $X_{t}^{\left(K\right)}$. Similar to Theorem 3, we can show that the corresponding error bound is of the same order of magnitude as


$$d[G^{-\omega_{1}}+G^{(\gamma +dK)/(2\gamma )+4\omega_{2}}\;T^{-\gamma /\{2\gamma +dK\}}\log^{3}\;(TG)].$$

We note that this upper bound depends on the order *K* only through the exponents of *G* and *T*.

### 4.2 Consistency of the proposed test

Given the error bound of the MDN estimator, we now establish the consistency, i.e. the size and power properties of our proposed test. We first show the bias of $\hat{S}(q,\mu ,\nu )$ converges at a faster rate than the forward and backward generators.

Assumption 4 Suppose ${\hat{\;f}}_{X_{t+1}|X_{t}}^{\left(\ell \right)}$ and ${\hat{\;f}}_{X_{t}|X_{t+1}}^{\left(\ell \right)}$ converge at a rate of $O(T^{-\kappa_{0}})$ for some $\kappa_{0}>0$. More specifically, suppose

$$\begin{array}{rl} & \sqrt{E\int_{x,y}|{\hat{\;f}}_{X_{t+1}|X_{t}}^{\left(\ell \right)}(y|x)-f_{X_{t+1}|X_{t}}^{*}(y|x){|}^{2}dxdy}=O(T^{-\kappa_{0}}),\\ & \sqrt{E\int_{x,y}|{\hat{\;f}}_{X_{t}|X_{t+1}}^{\left(\ell \right)}(y|x)-f_{X_{t+1}|X_{t}}^{*}(y|x){|}^{2}dxdy}=O(T^{-\kappa_{0}}), \end{array}$$

where the expectation is taken with respect to ${\hat{\;f}}_{X_{t+1}|X_{t}}^{\left(\ell \right)}$ and ${\hat{\;f}}_{X_{t}|X_{t+1}}^{\left(\ell \right)}$.

Theorem 4 Suppose Assumption 4 holds. Then under the null hypothesis $H_{0}$,

$$\underset{q,\mu ,\nu }{\sup}\left|E\hat{S}(q,\mu ,\nu )\right|=O\left(\;f_{\max}T^{-2\kappa_{0}}\right),$$

where $f_{\max}=\underset{x}{\sup}\max_{1\leq t\leq T}f_{X_{t}}(x)$, and $f_{X_{t}}$ denotes the marginal density function of $X_{t}$.

We note that, when the marginal density functions are uniformly bounded, Theorem 4 formally verifies the faster convergence rate of the bias of $\hat{S}(q,\mu ,\nu )$.

Next, we establish the size property of the proposed test.

Assumption 5 Suppose the following conditions hold.

- The convergence rates for ${\hat{\;f}}_{X_{t+1}|X_{t}}^{\left(\ell \right)}$ and ${\hat{\;f}}_{X_{t}|X_{t+1}}^{\left(\ell \right)}$ are both $O(T^{-\kappa_{0}})$ for some $\kappa_{0}>1/4$.
- Suppose there exists some ϵ>0, such that the real and imaginary parts of $\left\{\;\exp\;(i\mu^{⊤}X_{t+q}\right)$  $-\varphi^{*}(\mu |X_{t+q-1})\}\{\;\exp\;(i\nu^{⊤}X_{t})-\psi^{*}(\nu |X_{t+1})\}$ have their variances greater than ϵ, for any μ,ν and q∈{0,…,Q}.
- Suppose $M=\kappa_{1}T^{\kappa_{2}}$ for some $\kappa_{1}>0,\kappa_{2}\geq 1/2$, and $Q\leq \max(\rho_{0}T,T-2)$ for some constant $0<\rho_{0}<1$.
- Suppose *B* grows polynomially fast with respect to *T*.

Assumption 5(i) requires the convergence rates of ${\hat{\;f}}_{X_{t+1}|X_{t}}^{\left(\ell \right)}$ and ${\hat{\;f}}_{X_{t}|X_{t+1}}^{\left(\ell \right)}$ to be $o(T^{-1/4})$, which allows us to derive the size property of the test based upon Theorem 3. This condition is reasonable. For instance, when the time series dimension *d* is fixed, this corresponds to requiring that γ>d/2 for Example 1, and γ>2.69d for Example 2. Meanwhile, we may also relax this condition, by using the theory of higher-order influence functions (Robins et al., 2017). Assumption 5(ii) is a technical condition to help simplify the theoretical analysis. Essentially, it is used to guarantee that the diagonal elements of the asymptotic covariance matrix are bounded away from zero. When the fitted MDN is consistent, it follows that the diagonal elements of the estimated covariance matrix are bounded away from zero as well, with probability tending to 1. This allows us to apply Theorem 1 of Chernozhukov et al. (2017) to establish the size property. This condition automatically holds when the conditional density functions $f_{X_{t+1}|X_{t}}^{*}$, $f_{X_{t}|X_{t+1}}^{*}$, $\|\mu_{b}{\|}_{2}$s and $\|\nu_{b}{\|}_{2}$s are uniformly bounded away from zero. Meanwhile, if we truncate the diagonal elements of the estimated covariance matrix from below by some small positive constant, then this condition is not needed, and the subsequent test remains valid to control the type-I error. Finally, Assumption 5(iii) and (iv) impose some requirements on the parameters M,Q and *B*. In particular, *B* is allowed to diverge with *T*. Therefore, the classical weak convergence theorem is not applicable to show the asymptotic equivalence between the distribution of the test statistic and that of the bootstrap samples given the data. To overcome this issue, we employ the high-dimensional martingale central limit theorem recently developed by Belloni and Oliveira (2018).

Theorem 5 Suppose Assumptions 1 and 5 hold. Then, as T→∞, $P(\hat{S}>{\hat{c}}_{\alpha })=\alpha +o(1)$ under the null hypothesis.

Next, we establish the power property of the proposed test.

Assumption 6 Suppose the following conditions hold.

- Suppose $\underset{q,\mu ,\nu }{\sup}S_{0}(q,\mu ,\nu )\gg T^{-1/2}$  $\log^{1/2}\;(T)$, where $S_{0}(q,\mu ,\nu )\;=|E\{\;\exp\;(i\mu^{⊤}X_{t+q})-\varphi^{*}(\mu |X_{t+q-1})\}\{\;\exp\;(i\nu^{⊤}X_{t})-\psi^{*}(\nu |X_{t+1})\}|$.
- Suppose $B=\kappa_{3}T^{\kappa_{4}}$ for some $\kappa_{3}>0,\kappa_{4}\geq 1/2$.

Assumption 6(i) measures the degree to which the alternative hypothesis deviates from the null. This is because, for q=1,…,Q, the quantity


(16) $$\underset{\;f,g}{\sup}\left|E\left[\;f(X_{t+q})-E\{f(X_{t+q})|X_{t+q-1}\}\right]\left[g(X_{t})-E\{g(X_{t})|X_{t+1}\}\right]\right|$$

measures the weak conditional dependence between $X_{t+q}$ and $X_{t}$ given $X_{t+q-1}$ and $X_{t+1}$ (Daudin, 1980). Here, the supremum is taken with respect to the class of all squared integrable functions of *X*, i.e. $L_{2}(X)$. According to the Weierstrass approximation theorem, the class of trigonometric polynomials are dense in $L_{2}(X)$. As such, (16) is equal to zero if and only if $\underset{\mu ,\nu }{\sup}S_{0}(q,\mu ,\nu )=0$. Therefore, $\underset{q,\mu ,\nu }{\sup}S_{0}(q,\mu ,\nu )$ measures the degree to which the alternative hypothesis deviates from the null, and we require it to be lower bounded. Assumption 6(ii) is mild as *B* is user-specified.

Theorem 6 Suppose Assumptions 1, 5(i) to (iii), and 6 hold. Then, as T→∞, $P(\hat{S}>{\hat{c}}_{\alpha })\to 1$ under the alternative hypothesis.

We remark that our proposed test is built on weak conditional independence, and thus is not consistent against *all* alternatives. There are cases when (16) equals zero but (2) does not hold, since weak conditional independence does not fully characterise conditional independence. In those cases, our test becomes powerless. A possible remedy is to consider an alternative doubly robust test statistics based on


$$\begin{array}{r} E\left[\left\{\exp\;(i\mu^{⊤}X_{t+q})-\varphi^{*}(\mu |X_{t+q-1})\right\}\left\{\exp\;(i\nu^{⊤}X_{t})-\psi^{*}(i\nu^{⊤}X_{t+1})\right\}\exp\;\{i(X_{t+1}^{⊤},\ldots ,X_{t+q-1}^{⊤})\}\omega_{q}\right]. \end{array}$$

The above expectation equals zero for any q≥2, $\mu ,\nu \in R^{d}$, and $\omega_{q}\in R^{d(q-1)}$, and the resulting supremum type test is consistent against all alternative hypotheses. However, it is computationally more expensive, since a large number of Monte Carlo samples $\{(\mu_{b},\nu_{b},\omega_{q,b}){\}}_{b}$ are needed to approximate the supremum over the space of $R\times R\times R^{d(q-1)}$ when *q* is large. In addition, our numerical analysis finds this test less powerful compared to our proposed test. This agrees with the observation in the literature that, even though the test based on weak conditional dependence is not consistent against all alternatives, it may benefit from a simple procedure, and thus a better power property (Li & Fan, 2020).

We also note that Theorems 5 and 6 have suggested some theoretical choices of the parameters L,B,M,Q. In practice, we recommend to set *L* fixed, and set *M* to be proportional to the sample size. Besides, we choose a large value for *Q* that is proportional to *T*, and also choose a large *B*. We discuss their empirical choices in the next section.

## 5 Simulations

We study the empirical performance of our proposed test through simulations. We consider three different Markov time series models, each with order K=3, dimension d=3, and varying length T={500,1000,1500,2000}. We apply the proposed sequential testing procedure for k=1,2,…,5, and report the percentage of times out of 500 data replications when the null hypothesis is rejected. When k<K, this percentage reflects the empirical power of the test, and when k≥K, it shows the empirical size.

We consider a linear type VAR model, a nonlinear type threshold model, and a nonlinear type GARCH model, all of which are commonly used in the time series literature (e.g. Auestad & Tjøstheim, 1990; Cheng & Tong, 1992; Tschernig & Yang, 2000).

**Model 1**: VAR model


$$\begin{array}{rl} A_{1} & =\left(\begin{array}{ccc} 0.5 & -0.2 & -0.2\\ -0.2 & 0.5 & -0.2\\ -0.2 & -0.2 & 0.5 \end{array}\right),\;A_{2}=\left(\begin{array}{ccc} -0.5 & 0.2 & 0.2\\ 0.2 & -0.5 & 0.2\\ 0.2 & 0.2 & -0.5 \end{array}\right),\;A_{3}=\left(\begin{array}{ccc} 0.4 & -0.1 & -0.1\\ -0.1 & 0.4 & -0.1\\ -0.1 & -0.1 & 0.4 \end{array}\right),\\ X_{t} & =A_{1}X_{t-1}+A_{2}X_{t-2}+A_{3}X_{t-3}+\varepsilon_{t}, \end{array}$$

where $X_{t},\varepsilon_{t}\in R^{3}$, and $\varepsilon_{t,1},\varepsilon_{t,2},\varepsilon_{t,3}\overset{iid}{∼}\mathrm{Normal}(0,0.5)$.

**Model 2**: Threshold model


$$\begin{array}{rl} & A_{1}=\left(\begin{array}{ccc} 0.5 & -0.2 & -0.2\\ -0.2 & 0.5 & -0.2\\ -0.2 & -0.2 & 0.5 \end{array}\right),\;A_{2}=\left(\begin{array}{ccc} -0.5 & 0.2 & 0.2\\ 0.2 & -0.5 & 0.2\\ 0.2 & 0.2 & -0.5 \end{array}\right),\;A_{3}=\left(\begin{array}{ccc} 0.4 & -0.1 & -0.1\\ -0.1 & 0.4 & -0.1\\ -0.1 & -0.1 & 0.4 \end{array}\right),\\ & B_{1}=\left(\begin{array}{ccc} 0.3 & -0.1 & -0.1\\ -0.1 & 0.3 & -0.1\\ -0.1 & -0.3 & 0.3 \end{array}\right),\;B_{2}=\left(\begin{array}{ccc} -0.3 & 0.1 & 0.1\\ 0.1 & -0.3 & 0.1\\ 0.1 & 0.1 & -0.3 \end{array}\right),\;B_{3}=\left(\begin{array}{ccc} 0.25 & -0.05 & -0.05\\ -0.05 & 0.25 & -0.05\\ -0.05 & -0.05 & 0.25 \end{array}\right),\\ & \left\{ \begin{array}{cc} X_{t}=A_{1}X_{t-1}+A_{2}X_{t-2}+A_{3}X_{t-3}+\epsilon_{t} & \text{ if }\sum_{\;j=1}^{3}X_{t-1,j}\leq 0,\\ X_{t}=B_{1}X_{t-1}+B_{2}X_{t-2}+B_{3}X_{t-3}+\epsilon_{t} & \text{ if }\sum_{\;j=1}^{3}X_{t-1,j}>0, \end{array}\right. \end{array}$$

where $X_{t},\varepsilon_{t}\in R^{3}$, and $\varepsilon_{t,1},\varepsilon_{t,2},\varepsilon_{t,3}\overset{iid}{∼}\mathrm{Normal}(0,0.5)$.

**Model 3**: Multivariate ARCH model


$$\left\{ \begin{array}{rl} & X_{t}=A{\tilde{X}}_{t},\;{\tilde{X}}_{t}=({\tilde{X}}_{t,1},{\tilde{X}}_{t,2},{\tilde{X}}_{t,3}{)}^{⊤},\;{\tilde{X}}_{t,j}=h_{t,j}^{\frac{1}{2}}\varepsilon_{t,j},\;j=1,2,3\\ & h_{t,1}=0.1+0.6{\tilde{X}}_{t-1,1}^{2}+0.35{\tilde{X}}_{t-3,1}^{2}\\ & h_{t,2}=0.2+0.8{\tilde{X}}_{t-1,2}^{2}+0.05{\tilde{X}}_{t-2,2}^{2}+0.1{\tilde{X}}_{t-3,2}^{2}\\ & h_{t,3}=0.1+0.3{\tilde{X}}_{t-1,3}^{2}+0.65{\tilde{X}}_{t-3,3}^{2} \end{array}\right.A=\left(\begin{array}{ccc} 1 & 0.2 & 0.2\\ 0.2 & 1 & 0.2\\ 0.2 & 0.2 & 1 \end{array}\right),$$

where $X_{t},\varepsilon_{t}\in R^{3}$, and $\varepsilon_{t,1},\varepsilon_{t,2},\varepsilon_{t,3}\overset{iid}{∼}\mathrm{Normal}(0,0.5)$.

We apply the proposed test. For the hyper-parameters, we propose to select the number of mixture components *G* using cross-validation, as its choice is important to the empirical performance. When *G* is small, the fitted MDN model may suffer from a large bias, leading to an inflated type-I errors, whereas when *G* is large, the model may be overfitted, yielding a more variable test statistic. For the number of pairs *B*, a larger value of *B* generally improves the power of the test, but also increases the computational cost. We thus fix it at B=1000 to achieve a trade-off between the power and the computational cost. For the rest of parameters, including the number of data chunks *L*, the number of pseudo samples *M*, and the largest number of lags *Q*, we conduct a sensitivity analysis in Section B.1 of the Online Supplementary Material, Appendix. We find that the proposed test is not overly sensitive to the choice of these parameters, as long as they are in a reasonable range. We thus set L=3, M=100, and Q=10 in our numerical studies. For MDN, we fix the number of layers H=1, and vary the number of nodes *U* per hidden layer to vary the total number of parameters, and correspondingly the overall complexity of MDN. We carry out another sensitivity analysis for *U* in Online Supplementary Material, Section B.1, and again find a similar performance of the test in a range of values of *U*, so we fix U=20 for the first two models, and U=40 for the last model, as the last one is more complex. We estimate the parameters of MDN through maximum likelihood, where the derivative of the likelihood function with respect to each parameter is derived and the back-propagation is employed. In our implementation, we employ the Adam algorithm (Kingma & Ba, 2015), and use Python and Tensorflow (Dillon et al., 2017). We publish our code on GitHub.^1^

We compare our proposed test with two baseline tests for the Markov property, including the test by Chen and Hong (2012), which used LPFs to estimate the CCFs, and a version of the random forest-based test by Shi et al. (2020), which was designed for reinforcement learning, and is modified and adapted to our setting. In addition, Chen and Hong (2012) suggested two methods to compute the *p*-value for their test. The first method estimates the asymptotic variance of the test and uses a normal approximation. The second method employs bootstrap. In our settings, we find that the bootstrap procedure is extremely slow for a large *T*. As such, we calculate the *p*-value based on the normal approximation.

Table 1 reports the empirical rejection rate of each test under the significance level α=0.05, aggregated over 500 data replications. It can be seen that the proposed test effectively controls the type-I error when k≥3, and is very powerful when k<3. To the contrary, both the two baseline tests suffer from inflated type-I errors for large *T*. For instance, when T≥1000, the type-I error of the test of Chen and Hong (2012) exceeds 0.09 in all cases. This is probably due to that the LPR tends to suffer with a larger dimension in the multivariate setting (Taylor & Einbeck, 2013). The test of Shi et al. (2020) has considerably large type-I errors when applied to the multivariate ARCH model. This is likely due to the fact that their test was not designed for time series data.

**Table 1.** Percentage of times out of 500 data replications when the null hypothesis is rejected under the significance level α=0.05

	T=500			T=1000			T=1500		
*k*	MDN	RF	LPF	MDN	RF	LPF	MDN	RF	LPF
Model 1: VAR model									
1	0.952	0.980	0.010	1.000	1.000	0.280	1.000	1.000	0.722
2	0.258	0.508	0.016	0.856	0.954	0.116	0.992	1.000	0.204
3	0.052	0.422	0.020	0.042	0.762	0.132	0.060	0.934	0.200
4	0.042	0.060	0.020	0.044	0.048	0.112	0.058	0.048	0.200
5	0.056	0.052	0.032	0.044	0.050	0.134	0.048	0.044	0.220
Model 2: Threshold model									
1	0.614	0.704	0.000	0.998	0.998	0.168	1.000	1.000	0.484
2	0.160	0.246	0.028	0.716	0.692	0.122	0.976	0.966	0.278
3	0.062	0.126	0.026	0.056	0.128	0.118	0.066	0.234	0.170
4	0.040	0.070	0.028	0.036	0.042	0.112	0.048	0.052	0.188
5	0.060	0.068	0.030	0.056	0.038	0.096	0.034	0.038	0.146

	T=1000			T=1500			T=2000		
*k*	MDN	RF	LPF	MDN	RF	LPF	MDN	RF	LPF
Model 3: Multivariate ARCH model									
1	0.368	0.842	0.244	0.648	0.966	0.552	0.846	1.000	0.840
2	0.332	0.826	0.240	0.642	0.960	0.528	0.838	1.000	0.734
3	0.064	0.398	0.098	0.044	0.520	0.210	0.058	0.794	0.284
4	0.042	0.286	0.090	0.050	0.356	0.202	0.054	0.554	0.236
5	0.064	0.252	0.094	0.058	0.328	0.154	0.064	0.484	0.228

*Note*: The true order of the Markov model is K=3 in all examples. Three methods are compared: our proposed test (MDN), Shi et al. (2020)’s method (RF), and Chen and Hong (2012)’s method (LPF).

Finally, we report the computation time of the proposed test. We ran all simulations on savio2 htc node of the UC Berkeley Computing Platform, with 12 CPUs and 128 GB RAM, and it took around 2 min on average for a single data replication. We also run an example on a regular laptop computer with a single CPU and 8 GB memory RAM, and it took around 20 min on average for one data replication.

## 6 Real data applications

We illustrate our method with three datasets: the temperature dataset (Example 1 of Chang et al., 2018), the PM2.5 dataset (Example 4 of Chang et al., 2018), and the diabetes dataset (Marling & Bunescu, 2018).

The first dataset consists of the monthly temperature of seven cities in Eastern China from January 1954 to December 1998. To remove the seasonal trend, we subtract the average across the same month of the year. This ensures that the resulting time series is stationary. The resulting time series has dimension d=7 and length T=528.

The second dataset consists of the daily average PM2.5 concentration readings, in the logarithmic scale, at 74 monitoring stations in Beijing and nearby areas of China from January 1, 2015 to December 31, 2016. PM2.5 refers to the mix of solid and liquid particles whose diameters are smaller than 2.5 micrometers, and is a key measure of air quality and pollution. We again subtract the average across the same day of the year. The resulting times series has dimension d=74 and length T=731.

The third dataset consists of measurements, recorded every 5 min, involving blood glucose level, meal, exercise and insulin treatment from six patients with type-I diabetes over eight weeks. We divide each day into 1-h intervals, and compute the average blood glucose level, the carbohydrate estimate for the meal, the exercise intensity, and the amount of insulin received during the 1-h interval. For each patient, the resulting time series has dimension d=4 and length T=1100.

We note that the third data example is different from the other two examples as well as the setting of our problem in several ways. First, for each $d_{0}$-dimensional time series, there are N=6 replications corresponding to six patients. Second, for the $d_{0}=4$ variables, it is of interest to test the Markov property for three of them, but not the insulin amount, because the amount of insulin is determined by the patients themselves. In addition, the insulin amount should be included in the conditioning set, because it directly affects the blood glucose level. Finally, for the carbohydrate estimate of the meal and the exercise intensity, a good portion of the measurements are zero, because no meal or exercise was taken in those time intervals. We modify the test in Algorithm 1 to accommodate these differences. Specifically, in Step 1, to tackle multiple replications, instead of splitting a single time series into multiple chunks, we now randomly split *N* replications into multiple chunks of similar sizes. In Step 2, to test the Markov property of a subset of variables of the multivariate time series, instead of estimating ${\hat{\;f}}_{X_{t}|X_{t-1}}^{\left(\ell \right)}$, we now estimate the forward generator ${\hat{\;f}}_{{\tilde{X}}_{t}|X_{t-1}}^{\left(\ell \right)}$, where ${\tilde{X}}_{t}$ only includes those variables to test about. Meanwhile, we still estimate the backward generator ${\hat{\;f}}_{X_{t}|X_{t-1}}^{\left(\ell \right)}$ as before. Also in Step 2, to tackle the issue that some observed time series involve many zeros, we fit a logistic regression to estimate the conditional densities, while we still use MDN for other continuous time series. The rest of steps remain essentially the same as in Algorithm 1.

We apply the proposed test, as well as the two alternative tests of Chen and Hong (2012) and Shi et al. (2020), for k=1,2,…,12 sequentially, to the three datasets. Table 2 reports the corresponding *p*-values. For both the temperature and PM2.5 datasets, our test suggests the Markov property holds. This result is consistent with the findings in the literature, as a simple vector autoregressive model of order 1 is sufficient to model these high-dimensional datasets (see, e.g. Chang et al., 2018). For the diabetes data, the test suggests the order of the Markov model is 4, which is consistent with the finding of Shi et al. (2020). By contrast, the test of Chen and Hong (2012) yields a large *p*-value when k=2 then a very small *p*-value when k=4 for the diabetes dataset. The test of Shi et al. (2020) tends to select a large value of *k* for both the temperature dataset and the PM2.5 dataset.

**Table 2.** The *p*-values of the sequential tests for k=1,2,…,12 for the three datasets: the temperature data, the PM2.5 data, and the diabetes data, by the three methods: our proposed test (MDN), Shi et al. (2020)’s method (RF), and Chen and Hong (2012)’s method (LPF)

Order *k*	1	2	3	4	5	6	7	8	9	10	11	12
MDN												
Temperature data	0.110	0.187	0.371	0.591	0.454	0.282	0.186	0.049	0.206	0.117	0.780	0.027
PM2.5 data	0.394	0.365	0.259	0.467	0.706	0.140	0.288	0.437	0.312	0.168	0.355	0.470
Diabetes data	0	0.010	0.030	0.240	0.243	0.421	0.436	0.485	0.360	0.338	0.485	0.411
RF												
Temperature data	0	0.097	0.154	0.063	0.023	0.052	0.052	0.026	0.025	0.037	0.019	0.031
PM2.5 data	0.052	0.004	0.067	0.047	0.056	0.044	0.029	0.006	0.052	0.119	0.137	0.119
Diabetes data	0	0.001	0.003	0.097	0.084	0.092	0.066	0.069	0.091	0.103	0.124	0.096
LPF												
Temperature data	0.805	0.847	0.513	0.807	0.250	0.754	0.705	0.144	0.448	0.214	0.948	0.315
PM2.5 data	0.201	0.645	0.522	0.336	0.493	0.265	0.245	0.035	0.676	0.091	0.857	0.491
Diabetes data	0	0.225	0.036	0.001	0.915	0.131	0.668	0.866	0.135	0.068	0.935	0.013

## Supplementary Material

qkad064_Supplementary_Data

## Data Availability

The monthly temperature dataset is openly available from our GitHup repository markov_test at https://github.com/yunzhe-zhou/markov˙test/tree/main/data. The PM 2.5 dataset is openly available from the UCI machine learning repository at https://archive.ics.uci.edu/ml/datasets/Beijing+PM2.5+Data. The OhioT1DM dataset is available from the Ohio University at http://smarthealth.cs.ohio.edu/OhioT1DM-dataset.html. Access to the last dataset is subject to approval and a data sharing agreement.

## Footnotes

https://github.com/yunzhe-zhou/markov_test.
