# Supplementary material for: Testing for the Markov property in time series via deep conditional generative learning
Source: J R Stat Soc Series B Stat Methodol. 2023 Jun 23;85(4):1204–22. doi: 10.1093/jrsssb/qkad064 (PMC10541293; doi:10.1093/jrsssb/qkad064)
Supplement: qkad064_Supplementary_Data [file qkad064_supplementary_data.pdf]

Supplement for “Testing for the Markov Property in Time Series via Deep Conditional Generative Learning”

Yunzhe Zhou[‡], Chengchun Shi[†], Lexin Li[‡], and Qiwei Yao [†]

[†]*London School of Economics and Political Science, London, UK*

[‡]*University of California at Berkeley, California, USA*

In this supplement, we first present the proofs of all the theoretical results in the paper, and some useful auxiliary lemmas. We then present some additional numerical results.

A Proofs

A.1 Proof of Lemma 1

Denote a G-partition of the interval $[-C_1, C_1]$ as $a_1 = -C_1, a_2 = -C_1 + 2C_1/G, \ldots, a_g = -C_1 + 2C_1(g-1)/G, \ldots, a_{G+1} = C_1$. Since the support of g belongs to the interval $[-C_1, C_1]$,

$$f^*(y|x) = \int_{-C_1}^{C_1} g(y_0|x)\phi_\sigma(y-y_0)dy_0 = \sum_{g=1}^{G}\int_{a_g}^{a_{g+1}} g(y_0|x)\phi_\sigma(y-y_0)dy_0. \tag{S1}$$

It follows from Taylor's theorem that

$$\phi_\sigma(y-y_0) = \phi_\sigma(y-a_g) - \phi_\sigma^{'}(y-\eta_{g,y_0})(y_0-a_g) \leq \phi_\sigma(y-a_g) + c(y_0-a_g),$$

for some $\eta_{g_{y_0}}$ that lies between y_0 and a_g, where the constant $c = 2C_1/\sqrt{2\pi\sigma^3}$ is uniform in (g, y_0). This, together with (S1), yields that

$$f^*(y|x) \leq \sum_{g=1}^{G}\int_{a_g}^{a_{g+1}} g(y_0|x)dy_0\ \phi_\sigma(y-a_g) + c\sum_{g=1}^{G}\int_{a_g}^{a_{g+1}} g(y_0|x)(y_0-a_g)dy_0.$$

Given that g is uniformly bounded away from infinity, the second term on the right-hand-side is of the order of magnitude $O(G^{-1})$. This completes the proof of Lemma 1. □

A.2 Proof of Theorem 1

The proof is similar to that of Theorem 1 of Shi et al. (2020), and we outline the key steps here.

Specifically, if the conditional independence (2) holds, it follows that

$$\varphi^*(\mu|X_{t+q-1})\mathbb{E}[\exp(i\nu^\top X_t)|\{X_j\}_{t<j\leq t+q-1}] = \mathbb{E}\left[\exp(i\mu^\top X_{t+q} + i\nu^\top X_t)|\{X_j\}_{t<j\leq t+q-1}\right],$$

almost surely, for any $t > 0$, $q \geq 2$ and the vectors $\mu, \nu \in \mathbb{R}^d$.

When (3) holds, we begin with $q = 2$. We have that

$$\varphi^*(\mu|X_{t+1})\mathbb{E}\{\exp(i\nu^\top X_t)|X_{t+1}\} = \mathbb{E}\left[\exp(i\mu^\top X_{t+2} + i\nu^\top X_t)|X_{t+1}\right],$$

almost surely, for any $t > 0$ and $\mu, \nu \in \mathbb{R}^d$. By Lemma 3 of Shi et al. (2020), we obtain that

$$X_{t+2} \perp\!\!\!\perp X_t \mid X_{t+1}, \quad \text{for any } \ t > 0.$$

For $q = 3$, by (3), we have that

$$\varphi^*(\mu|X_{t+2})\mathbb{E}\{\exp(i\nu^\top X_t)|\{X_j\}_{t<j\leq t+2}\} = \mathbb{E}\left[\exp(i\mu^\top X_{t+q} + i\nu^\top X_t)|\{X_j\}_{t<j\leq t+2}\right], \quad \text{(S2)}$$

for any $t > 0, \mu, \nu \in \mathbb{R}^d$. For any $v \in \mathbb{R}^d$, multiplying both sides of (S2) by $\exp\left(iv^\top X_{t+1}\right)$ and taking the expectation with respect to X_{t+1} conditional on X_{t+2}, we obtain that

$$\begin{aligned}&\mathbb{E}\left\{\exp\left(i\mu^\top X_{t+3}\right) \mid X_{t+2}\right\}\mathbb{E}\left\{\exp\left(iv^\top X_t + i\nu^\top X_{t+1}\right) \mid X_{t+2}\right\}\\ &= \mathbb{E}\left\{\exp\left(i\mu^\top X_{t+3} + iv^\top X_t + i\nu^\top X_{t+1}\right) \mid X_{t+2}\right\}.\end{aligned}$$

By Lemma 3 of Shi et al. (2020) again, we obtain that

$$X_{t+3} \perp\!\!\!\perp X_t, X_{t+1} \mid X_{t+2}, \quad \text{for any } \ t > 0.$$

Similarly, we can show that, for any $q > 4$,

$$X_{t+q} \perp\!\!\!\perp \{X_j\}_{t\leq j<t+q-1} \,|X_{t+q-1}, \quad \text{for any } \ t > 0.$$

This completes the proof of Theorem 1. □

A.3 Proof of Theorem 2

When $\varphi = \varphi^*$, we have that,

$$\mathbb{E}\left[\exp(i\mu^\top X_{t+q+1}) - \varphi^*(\mu|X_{t+q})|\{X_j\}_{j\leq t+q}\right] = 0,$$

under the Markov property. Therefore, Equation (5) in Theorem 2 holds. In addition, we have $X_{t-1} \perp\!\!\!\perp \{X_j\}_{j>t}|X_t$ for any $t > 1$. When $\psi = \psi^*$, we can similarly show that,

$$\mathbb{E}\left[\exp(i\nu^\top X_{t-1}) - \psi^*(\nu|X_t)|\{X_j\}_{j>t}\right] = 0.$$

The doubly-robustness property thus follows. This completes the proof of Theorem 2. □

A.4 Proof of Theorem 3

We begin with some definitions. Let $\mathcal{F}_{\text{DNN}}$ denote the deep neural network for modeling each unit of MDN. The total number of parameters of $\mathcal{F}_{\text{DNN}}$ is W, and the number of hidden layers is H. For a given conditional density estimator $\widehat{f}$, define the following norms,

$$\|f\|_\infty = \sup_{x,y} |f(y|x)|, \qquad \|\widehat{f} - f^*\|_n = \sqrt{\frac{1}{T}\sum_{i=1}^{T} |\widehat{f}(y_i|x_i) - f^*(y_i|x_i)|^2}.$$

where $x_i = X_i$ and $y_i = X_{i+1}$. Since L is fixed, the convergence rate of $\widehat{f}^{(\ell)}_{X_{t+1}|X_t}$ is the same as that of $\widehat{f}_{X_{t+1}|X_t}$, which is trained based on the entire dataset. We thus focus on establishing the upper error bound for $\widehat{f}_{X_{t+1}|X_t}$ in the rest of the proof.

We divide the proof into three major steps. In Step 1, we temporarily ignore the dependence over time and apply the machinery of Farrell et al. (2021) to derive the error bounds,

$$\begin{aligned}\mathbb{E}_T\left(\log f^* - \log f_T\right) \le& \frac{1}{2C^2}\left\{C_2' G^{4\omega_2}\epsilon + O(G^{-\omega_1})\right\}^2 \\ &+ \left\{C_2' G^{4\omega_2}\epsilon + O(G^{-\omega_1})\right\}\sqrt{\frac{2\widetilde{r}}{C^2(T-1)}} + \frac{7AG^{\omega_2}\widetilde{r}}{C(T-1)},\end{aligned} \tag{S3}$$

$$\begin{aligned}(\mathbb{E} - \mathbb{E}_T)(\log f^* - \log \widehat{f}) \le& 3C_4 r_0 G^{\omega_2}\sqrt{\frac{G\cdot \text{Pdim}(\mathcal{F}_{\text{DNN}})}{T}\log T} \\ &+ 2r_0 G^{\omega_2}\sqrt{\frac{\widetilde{r}}{C^2 T}} + \frac{23AG^{\omega_2}\widetilde{r}}{CT}\end{aligned} \tag{S4}$$

under the i.i.d. setting. In this step, compared to Farrell et al. (2021), our main contribution lies in analyzing a new deep generative learning model architecture.

We further divide this step into three sub-steps.

In Step 1.1, we show that the MDN model satisfies Condition (2.1) of Farrell et al. (2021). Then similar to Farrell et al. (2021), we decompose the error bound into the sum of the approximation error and the estimation error, i.e.,

$$\mathbb{E}_T\left(\log f^*_{X_{t+1,1}|X_t} - \log f_{T,X_{t+1,1}|X_t}\right), \quad \text{and} \quad (\mathbb{E}-\mathbb{E}_T)\left(\log f^*_{X_{t+1,1}|X_t} - \log \widehat{f}_{X_{t+1,1}|X_t}\right),$$

where $X_{t+1,1}$ denotes the first element of X_{t+1}, and $f_T \in \mathcal{F}$ denotes the best MDN approximation whose explicit form is given in Lemma S2 in Section A.8. Here we consider the first element of X_{t+1} as an example of one dimension case. We will generalize the result to high-dimension case in Step 3. The expectation $\mathbb{E}$ is taken with respect to the stationary distribution of (X_t, X_{t+1}), and $\mathbb{E}_T$ denotes the empirical mean operator, i.e., $\mathbb{E}_T[g] = (T-1)^{-1}\sum_{t=1}^{T-1} g(X_{t+1}|X_t)$.

In Step 1.2, We obtain Lemma S2 in Section A.8, then combine it with the arguments in Section A.1 of Farrell et al. (2021) to upper bound our approximation error.

In Step 1.3, we follow Section A.2 of Farrell et al. (2021) to upper bound the Rademacher complexity of the MDN function class, which in turn upper bounds our estimation error.

Next, in Step 2, we extend our results to the time-dependent setting based on Lemma S4 in Section A.8, which itself is based on Berbee's lemma (Berbee, 1979). We note that most existing deep learning theories are derived under the i.i.d. setting, whereas our arguments can potentially be used to help establish other deep learning theories under the time-dependent setting. This is another contribution of our theoretical analysis.

Finally, in Step 3, we combine the first two steps and establish the finite-sample convergence rate of $\widehat{f}_{X_{t+1}|X_t}$. We also note that our analysis applies to high-dimensional time series.

To simplify the notation, we omit the subscript $X_{t+1,1}|X_t$ in Steps 1and 2, and write $\widehat{f}_{X_{t+1,1}|X_t}$, $f^*_{X_{t+1,1}|X_t}$, $f_{T,X_{t+1,1}|X_t}$ as $\widehat{f}$, f^* and f_T, whenever there is no confusion.

Step 1.1 We start with the i.i.d. setting. We first check that Conditions (2.1) of Farrell et al. (2021) holds. Letting $A = C_2/\sqrt{2\pi}$, a uniform upper bound for any $f \in \mathcal{F}$ is given by,

$$\|f\|_\infty \leq \sum_{g=1}^{G} \frac{\alpha_g(x)}{\sqrt{2\pi}\sigma_g(x)} \leq \sum_{g=1}^{G} \frac{\alpha_g(x)C_2G^{\omega_2}}{\sqrt{2\pi}} = \frac{C_2G^{\omega_2}}{\sqrt{2\pi}} = AG^{\omega_2}. \tag{S5}$$

Under Assumption 3(i), the function class $\mathcal{F}$ is uniformly lower bounded. By Taylor's expansion, for any $f, g \in \mathcal{F}$, there exists a constant $C = \inf_{x,y} f(y \mid x) \wedge \inf_{x,y} g(y \mid x) > 0$, such that

$$|\log f(y|x) - \log g(y|x)| \ \ \leq \ \ \frac{1}{C}|f(y|x) - g(y|x)|.$$

Similar to (S5), we can obtain a lower bound for $|\log f(y|x) - \log g(y|x)|$. It follows that,

$$\frac{1}{2A^2G^{2\omega_2}}\mathbb{E}\{(f-f^*)^2\} \ \ \leq \ \ \mathbb{E}(\log f^*) - \mathbb{E}(\log f) \leq \frac{1}{2C^2}\mathbb{E}\{(f-f^*)^2\}.$$

where we use the Taylor expansion for the derivation of the inequalities. Specifically, we can show that there $\exists \widetilde{f} \in \mathcal{F}$ such that $\mathbb{E}(\log f^*) - \mathbb{E}(\log f) = \mathbb{E}[\frac{1}{2\widetilde{f}^2}(f-f^*)^2]$ since the expectation for the gradient of $\log f^*$ is 0. Then the inequalities can be derived according to the lower and upper bound of $\widetilde{f}$. Recall that $\widehat{f} = \arg\max_f \mathbb{E}_T \log f$. We apply the decomposition in Section A.1 of Farrell et al. (2021) and obtain that

$$\begin{aligned} \frac{1}{2A^2G^{2\omega_2}}\mathbb{E}\left\{(\widehat{f}-f^*)^2\right\} &\leq \mathbb{E}(\log f^*) - \mathbb{E}(\log \widehat{f}) \\ &\leq \mathbb{E}(\log f^*) - \mathbb{E}(\log \widehat{f}) - \mathbb{E}_T(\log f_T) + \mathbb{E}_T(\log \widehat{f}) \\ &= (\mathbb{E} - \mathbb{E}_T)(\log f^* - \log \widehat{f}) + \mathbb{E}_T(\log f^* - \log f_T). \end{aligned} \tag{S6}$$

Recall that f_T is the best MDN approximation.

Step 1.2 We upper bound the approximation error. Applying Lemma S2 in Section A.8 yields an upper error bound for $\epsilon = \|f^* - f_T\|_\infty$. This together with the Bernstein's inequality leads to an upper bound for $\mathbb{E}_T(\log f^* - \log f_T)$. Combining these two bounds, for any constant $\widetilde{r} > 0$, we obtain that, with probability at least $1 - e^{-\widetilde{r}}$,

$$\begin{aligned}\mathbb{E}_T\left(\log f^* - \log f_T\right) \leq \frac{1}{2C^2}\left\{C_2' G^{4\omega_2}\epsilon + O(G^{-\omega_1})\right\}^2 \\ + \left\{C_2' G^{4\omega_2}\epsilon + O(G^{-\omega_1})\right\}\sqrt{\frac{2\widetilde{r}}{C^2(T-1)}} + \frac{7AG^{\omega_2}\widetilde{r}}{C(T-1)}.\end{aligned} \tag{S7}$$

where the constant term $\frac{1}{2C^2}$ comes from the use of the inequality $\mathbb{E}(\log f^*) - \mathbb{E}(\log f) \leq \frac{1}{2C^2}\mathbb{E}\{(f - f^*)^2\}$ derived from Step 1.1. Note that we assume $\mathcal{F}$ is a bounded function class, and that f^* is upper bounded. As a result, the Bernstein inequality's conditions are satisfied.

Step 1.3 We upper bound the estimation error. Suppose there exists a constant $r_0 > 0$ such that $\|f - f^*\|_2 \leq r_0 G^{\omega_2}$. This holds true if we set $r_0 = 3A$, where A is defined in Step 1.1. It then follows that,

$$\mathbb{E}\left\{|\log(f) - \log(f^*)|^2\right\} \leq \frac{\mathbb{E}(f - f^*)^2}{C^2} \leq \frac{r_0^2 G^{2\omega_2}}{C^2}.$$

Applying Theorem 2.1 of Bartlett et al. (2005) to the function class $\mathcal{G} = \left\{g = \log f - \log f^* : f \in \mathcal{F}, \|f - f^*\|_2 \leq r_0 G^{\omega_2}\right\}$, we obtain that, with probability at least $1 - 2e^{-\widetilde{r}}$,

$$(\mathbb{E} - \mathbb{E}_T)(\log f^* - \log \widehat{f}) \leq 3\mathbb{E}_\eta R_n \mathcal{G} + \sqrt{\frac{2r_0^2 G^{2\omega_2}\widetilde{r}}{C^2(T-1)}} + \frac{23AG^{\omega_2}\widetilde{r}}{C(T-1)} \tag{S8}$$

where the empirical Rademacher complexity $\mathbb{E}_\eta R_n \mathcal{G}$ is defined by,

$$\mathbb{E}_\eta \sup_{g \in \mathcal{G}} \left\{\frac{1}{(T-1)} \sum_{t=1}^{(T-1)} \eta_t g(x_{t+1}|x_t)\right\},$$

in which $\{\eta_\tau\}_\tau$ are i.i.d. samples from the Rademacher distribution, and $\mathbb{E}_\eta$ is the expectation over this distribution.

Next, by contraction and Dudley's chaining properties (Mendelson, 2003), we obtain that, with probability at least $1 - e^{-\widetilde{r}}$,

$$\begin{aligned}\mathbb{E}_\eta R_n \mathcal{G} &= \mathbb{E}_\eta\left[R_n\left\{g : g = \log(f) - \log(f^*), f \in \mathcal{F}, \|f - f^*\|_{L_2} \leq r_0 G^{\omega_2}\right\}\right] \\ &\leq \frac{2}{C}\mathbb{E}_\eta\left[R_n\left\{f - f^*, f \in \mathcal{F}, \|f - f^*\|_{L_2} \leq r_0 G^{\omega_2}\right\}\right]\end{aligned}$$

$$
\begin{aligned}
&\leq \frac{2}{C}\mathbb{E}_\eta R_n\{f-f^*, f\in\mathcal{F}, \|f-f^*\|_n \leq 2r_0G^{\omega_2}\}\\
&\leq \frac{2}{C}\inf_{0<\alpha<2r_0G^{\omega_2}}\left\{4\alpha+\frac{12}{\sqrt{(T-1)}}\int_\alpha^{2r_0G^{\omega_2}}\sqrt{\log\mathcal{T}(\delta,\mathcal{F},\|\cdot\|_n)}d\delta\right\}\\
&\leq \frac{2}{C}\inf_{0<\alpha<2r_0G^{\omega_2}}\left\{4\alpha+\frac{12}{\sqrt{(T-1)}}\int_\alpha^{2r_0G^{\omega_2}}\sqrt{G\log\mathcal{T}(\frac{\delta}{C_3G^{3/2+3\omega_2}},\mathcal{F}_{\text{DNN}},\|\cdot\|_n)}d\delta\right\}\\
&\leq \frac{2}{C}\inf_{0<\alpha<2r_0G^{\omega_2}}\left\{4\alpha+\frac{12}{\sqrt{(T-1)}}\int_\alpha^{2r_0G^{\omega_2}}\sqrt{G\log\mathcal{T}(\frac{\delta}{C_3G^{3/2+3\omega_2}},\mathcal{F}_{\text{DNN}},\|\cdot\|_\infty)}d\delta\right\},
\end{aligned}
$$

where $\mathcal{F}$ is the MDN function class, and $\mathcal{F}_{\text{DNN}}$ is the DNN class defined at the beginning. The second last inequality is due to Lemma S3 in Section A.8, and α is some positive constant that will be specified later.

Next, applying Theorems 12.2 and 14.1 in Anthony et al. (1999), we further upper bound the entropy integral using the pseudo VC dimension of the neural network function class, denoted by $\text{Pdim}(\mathcal{F}_{\text{DNN}})$. Specifically, we have,

$$
\begin{aligned}
\mathbb{E}_\eta R_n\mathcal{G} &\leq \frac{2}{C}\inf_{0<\alpha<2r_0G^{\omega_2}}\left\{4\alpha+\frac{12}{\sqrt{(T-1)}}\int_\alpha^{2r_0G^{\omega_2}}\sqrt{G\cdot\text{Pdim}(\mathcal{F}_{\text{DNN}})\log\frac{eAT\cdot C_3G^{3/2+4\omega_2}}{\delta\cdot\text{Pdim}(\mathcal{F}_{\text{DNN}})}}d\delta\right\}\\
&\leq \frac{64r_0G^{\omega_2}}{C}\left\{\sqrt{\frac{G\cdot\text{Pdim}(\mathcal{F}_{\text{DNN}})}{T-1}(\log\frac{eAC_3G^{3/2+4\omega_2}}{r_0}+\frac{3}{2}\log T)}\right\},
\end{aligned}
$$

by setting $\alpha = 2r_0G^{\omega_2}\sqrt{G\cdot\text{Pdim}(\mathcal{F}_{\text{DNN}})/(T-1)}$.

Therefore, whenever $r_0 \geq 1/(T-1)$, and $T = O(G)$, there exists a constant $C_4 > 0$, such that, with probability at least $1-e^{-\widetilde{r}}$,

$$
\mathbb{E}_\eta R_n\mathcal{G} \leq C_4r_0G^{\omega_2}\sqrt{\frac{G\cdot\text{Pdim}(\mathcal{F}_{\text{DNN}})}{T}\log T} \tag{S9}
$$

Plugging (S9) into (S8), we obtain that,, with probability at least $1-6e^{-\widetilde{r}}$,

$$
\begin{aligned}
&(\mathbb{E}-\mathbb{E}_T)(\log f^*-\log\widehat{f})\\
&\leq 3C_4r_0G^{\omega_2}\sqrt{\frac{G\cdot\text{Pdim}(\mathcal{F}_{\text{DNN}})}{T}}\log T+2r_0G^{\omega_2}\sqrt{\frac{\widetilde{r}}{C^2T}}+\frac{23AG^{\omega_2}\widetilde{r}}{CT}
\end{aligned} \tag{S10}
$$

Step 2. The data observations in our setting are time-dependent. Nevertheless, thanks to the exponential β-mixing condition in Assumption 1, and the decoupling lemma, i.e., Lemma S4 in Section A.8, the estimation error is of the same order of magnitude as that under the i.i.d. setting, up to some logarithmic factors. More specifically, by Lemma S4, we can construct two

i.i.d. sequences, $\{U_{2i}^0\}_{i\geq 0}$ and $\{U_{2i+1}^0\}_{i\geq 0}$, such that $U_i^0 = (X_{ip+1}^0, X_{ip+2}^0, ..., X_{ip+p}^0)$ equals $U_i = (X_{ip+1}, X_{ip+2}, \dots, X_{ip+p})$ with probability at least $1-\beta(p)$. Without loss of generality, suppose $T-1$ is divisible by $2p$. Then with probability at least $1-T\beta(p)/p$, we have that,

$$\mathbb{E}_T(\log f^* - \log f_T) = \mathbb{E}_T^0(\log f^* - \log f_T), \tag{S11}$$

where $\mathbb{E}_T^0 \log f = (T-1)^{-1}\sum_{t=1}^{T-1} \log f(X_{t+1}^0 | X_t^0)$.

We next bound the approximation error (S11). By construction, the sequence $\{U_{2t}^0\}_{t\geq 0}$ is i.i.d., and so is $\{U_{2t+1}^0\}_{t\geq 0}$. We thus divide the original empirical sum into multiple pieces, and apply the empirical process theory to derive the upper error bound for each of these pieces separately.

More specifically, we have that,

$$\mathbb{E}_T^0\left(\log f^* - \log f_T\right) = \frac{1}{2p}\left\{\frac{1}{(T-1)/(2p)}\sum_{\tau=1}^{(T-1)/(2p)} \zeta_{p,2\tau-1} + \frac{1}{(T-1)/(2p)}\sum_{\tau=1}^{(T-1)/(2p)} \zeta_{p,2\tau}\right\} \tag{S12}$$

where $\zeta_{p,\tau} = \sum_{t=(\tau-1)p+1}^{\tau p}\left\{\log f^*(X_{t+1,1}^0|X_t^0) - \log f_T(X_{t+1,1}^0|X_t^0)\right\}$.

Following the same procedure of Step 1.2, for any constant $\widetilde{r} > 0$, we have that, with probability at least $1-e^{-\widetilde{r}}$,

$$\begin{aligned}\mathbb{E}_T^0\left(\log f^* - \log f_T\right) &\leq \frac{1}{2C^2}\left\{C_2' G^{4\omega_2}\epsilon + O(G^{-\omega_1})\right\}^2 \\ &\quad + \left\{C_2' G^{4\omega_2}\epsilon + O(G^{-\omega_1})\right\}\sqrt{\frac{4p\widetilde{r}}{C^2(T-1)}} + \frac{14pAG^{\omega_2}\widetilde{r}}{C(T-1)}.\end{aligned} \tag{S13}$$

Similarly, we have that, with probability at least $1-T\beta(p)/p$,

$$(\mathbb{E}-\mathbb{E}_T)(\log f^* - \log \widehat{f}) = (\mathbb{E}-\mathbb{E}_T^0)(\log f^* - \log \widehat{f}). \tag{S14}$$

This allows us to extend the result of Step 1.3 to obtain that, with probability at least $1-6e^{-\widetilde{r}}$,

$$\begin{aligned}&(\mathbb{E}-\mathbb{E}_T^0)(\log f^* - \log \widehat{f}) \\ &\leq 3C_4 r_0 G^{\omega_2}\sqrt{\frac{pG\cdot \mathrm{Pdim}(\mathcal{F}_{\mathrm{DNN}})}{T}\log T} + 2r_0 G^{\omega_2}\sqrt{\frac{p\widetilde{r}}{C^2 T}} + \frac{23pAG^{\omega_2}\widetilde{r}}{CT}\end{aligned} \tag{S15}$$

By Lemma 6 of Farrell et al. (2021), the pseudo VC dimension $\mathrm{Pdim}(\mathcal{F}_{\mathrm{DNN}})$ is of the order $O(WH\log W)$, where H is the number of layers in DNN. Using (S14), (S11), plugging (S13) and (S15) into (S6), and setting $p = 2/c_2 \log T$, we obtain that, with probability at least $1-$

$7e^{-\widetilde{r}} - T^{-1}c_1$,

$$\|\widehat{f} - f^*\|_2 \leq C_5 \log T \left[r_0 G^{\omega_2} \sqrt{\frac{GWH \log W}{T}} + r_0 G^{\omega_2} \sqrt{\frac{\widetilde{r}}{T}} + \frac{G^{\omega_2}\widetilde{r}}{T} + \epsilon^2 G^{8\omega_2} \right.$$
$$\left. + \epsilon G^{4\omega_2} \left\{ \sqrt{\frac{\widetilde{r}}{T}} + O(G^{-\omega_1}) \right\} \right] + O(G^{-2\omega_1}),$$

for some constant $C_5 > 0$, and c_1, c_2 are as defined in Assumption 1.

We next employ the techniques in Appendices A.2.3 and A.2.4 of Farrell et al. (2021) to recursively improve the upper bound r_0, which leads to that, for any constant $\widetilde{r} > 0$, with probability at least $1 - 7e^{-\widetilde{r}} - O(T^{-1})$,

$$\|\widehat{f} - f^*\|_2 \leq C_6 \left\{ \left(\sqrt{\frac{GWH \log W}{T}} \log^2 T + \log T \sqrt{\frac{\log\log T + \widetilde{r}}{T}} + \epsilon \log T \right) G^{4\omega_2} + O(G^{-\omega_1}) \right\},$$

for some constant $C_6 > 0$.

Recall that the approximation error ϵ of DNN depends on the model structure. By Lemma S2, there exists a DNN function class with the approximation error ϵ, such that

$$H \leq C_1\{\log(G/\epsilon) + 1\}, \quad W \leq C_1 G^{\frac{d}{\gamma}} \epsilon^{-\frac{d}{\gamma}} \{\log(G/\epsilon) + 1\},$$

for some constant $C_1 > 0$. Setting $\epsilon = T^{-\gamma/(2\gamma+d)}$, with probability at least $1 - 7e^{-\widetilde{r}} - O(T^{-1})$,

$$\begin{aligned} &\|\widehat{f} - f^*\|_2 \\ &\leq C_7 \left(\left[\sqrt{\frac{G^{\frac{\gamma+d}{\gamma}} \epsilon^{-\frac{d}{\gamma}} \{\log(G/\epsilon) + 1\}^3}{T}} \log^2 T + \log T \sqrt{\frac{\log\log T + \widetilde{r}}{T}} + \epsilon \log T \right] G^{4\omega_2} + O(G^{-\omega_1}) \right) \\ &\leq C_8 \left[\left\{ G^{\frac{\gamma+d}{2\gamma}} T^{-\frac{\gamma}{2\gamma+d}} \log^3(TG) + \log T \sqrt{\frac{\log\log T + \widetilde{r}}{T}} \right\} G^{4\omega_2} + O(G^{-\omega_1}) \right]. \end{aligned}$$

Setting $\widetilde{r} = \log T$, we obtain that, with probability at least $1 - O(T^{-1})$,

$$\|\widehat{f} - f^*\|_2 \leq O(G^{\frac{\gamma+d}{2\gamma} + 4\omega_2} T^{-\frac{\gamma}{2\gamma+d}} \log^3(TG)) + O(G^{-\omega_1}).$$

Step 3. According to the factorization rule, the true joint conditional density can be decomposed as: $f^*_{X_{t+1}|X_t} = \prod_{i=1}^d f^*_{X_{t+1,i}|X_t, X_{t+1,1}, \cdots, X_{t+1,i-1}}$. By Steps 1 and 2, we have obtained the upper bound for $\|\widehat{f}_{X_{t+1,1}|X_t} - f^*_{X_{t+1,1}|X_t}\|_2$. Following similar arguments, we can show that the same bound holds for $\|\widehat{f}_{X_{t+1,i}|X_t, X_{t+1,1}, \cdots, X_{t+1,i-1}} - f^*_{X_{t+1,i}|X_t, X_{t+1,1}, \cdots, X_{t+1,i-1}}\|_2$ for each $i = 1, \ldots, d$. Then applying triangle inequality iteratively, we have, with probability at least $1 - O(T^{-1})$,

$$\|\widehat{f}_{X_{t+1}|X_t} - f^*_{X_{t+1}|X_t}\|_2$$

$$
\begin{aligned}
&= \left\| \prod_{i=1}^{d} \widehat{f}_{X_{t+1,i}|X_t,X_{t+1,1},\cdots,X_{t+1,i-1}} - \prod_{i=1}^{d} f^*_{X_{t+1,i}|X_t,X_{t+1,1},\cdots,X_{t+1,i-1}} \right\|_2 \\
&\leq \left\| \prod_{i=2}^{d} \widehat{f}_{X_{t+1,i}|X_t,X_{t+1,1},\cdots,X_{t+1,i-1}} - \prod_{i=2}^{d} f^*_{X_{t+1,i}|X_t,X_{t+1,1},\cdots,X_{t+1,i-1}} \right\|_2 \\
&+ O(1) \left\| \widehat{f}_{X_{t+1,1}|X_t,X_{t+1,1},\cdots,X_{t+1,0}} - f^*_{X_{t+1,1}|X_t,X_{t+1,1},\cdots,X_{t+1,0}} \right\|_2 \\
&\leq \cdots \\
&\leq \sum_{i=1}^{d} O(1) \left\| \widehat{f}_{X_{t+1,i}|X_t,X_{t+1,1},\cdots,X_{t+1,i-1}} - f^*_{X_{t+1,i}|X_t,X_{t+1,1},\cdots,X_{t+1,i-1}} \right\|_2 \\
&\leq \left[O\left(G^{\frac{\gamma+d}{2\gamma}+4\omega_2} T^{-\frac{\gamma}{2\gamma+d}} \log^3(TG) \right) + O(G^{-\omega_1}) \right] d,
\end{aligned}
$$

where $O(1)$ denotes some positive constant. This completes the proof of Theorem 3. □

A.5 Proof of Theorem 4

The proof is similar to Step 1 of the Proof of Theorem 5 below, and is omitted. □

A.6 Proof of Theorem 5

The proof follows that of Theorem 3 of Shi et al. (2020), and is presented here for completeness. Essentially, we adopt similar arguments and apply to our setting of multivariate time series where we employ MDN instead of random forest to estimate $\widehat{\varphi}$ and $\widehat{\psi}$. Moreover, we remark that Theorem 5 is built upon Theorem 3, but cannot be directly deduced from Theorem 3. More specifically, Theorem 3 is about the rate of convergence of the MDN estimator, whereas Theorem 5 establishes the size property of the proposed test. While Theorem 5 requires the convergence rate result of Theorem 3, its proof also requires a number of additional techniques, including the high-dimensional Gaussian approximation theory (Chernozhukov et al., 2013), and the Neyman orthogonality (Chernozhukov et al., 2018). As a result, the proof of Theorem 5 is considerably different from that of Theorem 3.

Define

$$
\begin{aligned}
\Gamma^*(q,\mu,\nu) = \frac{1}{T-n-(q-1)(L-1)} \sum_{\ell=1}^{L-1} \sum_{t=1}^{n-q+1} &\{\exp(i\mu^\top X_{\ell\cdot n+t+q+1}) \\
&-\varphi^*(\mu|X_{\ell\cdot n+t+q})\}\{\exp(i\nu^\top X_{\ell\cdot n+t-1}) - \psi^*(\nu|X_{\ell\cdot n+t})\}.
\end{aligned}
$$

for any q, μ, ν. And Γ^*_R and Γ^*_I are the real and imaginary part of Γ^* respectively. Also denote

$$
\hat{\Gamma}(q,\mu,\nu) = \frac{1}{T-n-(q-1)(L-1)} \sum_{\ell=1}^{L-1} \sum_{t=1}^{n-q+1} \{ \exp(i\mu^\top X_{\ell\cdot n+t+q+1})
$$

$$-\hat{\varphi}(\mu|X_{\ell\cdot n+t+q})\}\{\exp(i\nu^\top X_{\ell\cdot n+t-1}) - \hat{\psi}(\nu|X_{\ell\cdot n+t})\}.$$

We divide the proof into three steps.

In step 1, we show that

$$\max_{b\in\{1,\cdots,B\}} \max_{q\in\{2,\cdots,Q\}} \sqrt{T-n-(q-1)(L-1)}|\widehat{\Gamma}(q,\mu_b,\nu_b) - \Gamma^*(q,\mu_b,\nu_b)| = o_p(\log^{-1/2}(T)),$$

which can be obtained by Lemma S5, by requiring $\widehat{\varphi}$ and $\widehat{\psi}$ to satisfy certain uniform convergence rates. Letting $S^* = \max_{b\in\{1,\cdots,B\}} \max_{q\in\{2,\cdots,Q\}} \sqrt{T-n-(q-1)(L-1)} \max(|\Gamma_R^*(q,\mu_b,\nu_b)|, |\Gamma_I^*(q,\mu_b,\nu_b)|)$, we can further obtain that,

$$\widehat{S} = S^* + o_p(\log^{-1/2}(T)). \tag{S16}$$

In Step 2, we show that, for any $z \in \mathbb{R}$ and any sufficiently small $\varepsilon > 0$,

$$\mathbb{P}(S^* \le z) \ge \mathbb{P}\left(\|N(0,V_0)\|_\infty \le z - \varepsilon\log^{-1/2}(T)\right) - o(1),$$
$$\mathbb{P}(S^* \le z) \le \mathbb{P}\left(\|N(0,V_0)\|_\infty \le z + \varepsilon\log^{-1/2}(T)\right) + o(1),$$

where the matrix V_0 is defined later. Combining it with (S16), we obtain that,

$$\begin{aligned}\mathbb{P}(\widehat{S} \le z) &\ge \mathbb{P}\left(\|N(0,V_0)\|_\infty \le z - 2\varepsilon\log^{-1/2}(T)\right) - o(1),\\ \mathbb{P}(\widehat{S} \le z) &\le \mathbb{P}\left(\|N(0,V_0)\|_\infty \le z + 2\varepsilon\log^{-1/2}(T)\right) + o(1).\end{aligned} \tag{S17}$$

In Step 3, we show that $\|V_0 - \widehat{V}\|_{\infty,\infty} = O((T)^{-c^{**}})$ for some $c^{**} > 0$ with probability tending to 1, where $\|\cdot\|_{\infty,\infty}$ denotes the element-wise max-norm. This, together with (S17), yields that, for any sufficiently small $\varepsilon > 0$, with probability tending to 1,

$$\mathbb{P}(\widehat{S} \le z) \ge \mathbb{P}\left(\|N(0,\widehat{V})\|_\infty \le z - 2\varepsilon\log^{-1/2}(T)|\widehat{V}\right) - o(1),$$
$$\mathbb{P}(\widehat{S} \le z) \le \mathbb{P}\left(\|N(0,\widehat{V})\|_\infty \le z + 2\varepsilon\log^{-1/2}(T)|\widehat{V}\right) + o(1),$$

where $\mathbb{P}(\cdot|\widehat{V})$ is the conditional probability given $\widehat{V}$. Setting $z = \widehat{c}_\alpha$, which is defined in Equation (13) of the paper, we obtain that, with probability tending to 1,

$$\begin{aligned}\mathbb{P}(\widehat{S} \le \widehat{c}_\alpha) &\ge \mathbb{P}\left(\|N(0,\widehat{V})\|_\infty \le \widehat{c}_\alpha - 2\varepsilon\log^{-1/2}(T)|\widehat{V}\right) - o(1),\\ \mathbb{P}(\widehat{S} \le \widehat{c}_\alpha) &\le \mathbb{P}\left(\|N(0,\widehat{V})\|_\infty \le \widehat{c}_\alpha + 2\varepsilon\log^{-1/2}(T)|\widehat{V}\right) + o(1),\end{aligned} \tag{S18}$$

We next show that, with probability tending to 1, the diagonal elements in $\widehat{V}$ are bounded away from zero, since the diagonal elements in V_0 are bounded away from zero given the conditions in

Theorem 5. It follows from Theorem 1 of Chernozhukov et al. (2017) that, conditioning on $\widehat{V}$, with probability tending to 1,

$$\mathbb{P}(\|N(0,\widehat{V})\|_\infty \le \widehat{c}_\alpha + 2\varepsilon \log^{-1/2}(T)|\widehat{V}) - \mathbb{P}(\|N(0,\widehat{V})\|_\infty \le \widehat{c}_\alpha - 2\varepsilon \log^{-1/2}(T)|\widehat{V}) \le o(1)\varepsilon \log^{1/2}(BQ)\log^{-1/2}(T),$$

where $o(1)$ denotes some positive constant that is independent of ε. Under the given conditions on B and Q, we obtain that, with probability tending to 1,

$$\mathbb{P}(\|N(0,\widehat{V})\|_\infty \le \widehat{c}_\alpha + 2\varepsilon \log^{-1/2}(T)|\widehat{V}) - \mathbb{P}(\|N(0,\widehat{V})\|_\infty \le \widehat{c}_\alpha - 2\varepsilon \log^{-1/2}(T)|\widehat{V}) \le C^*\varepsilon,$$

for some constant $C^* > 0$. Combining with (S18), we obtain that, with probability tending to 1,

$$\left|\mathbb{P}(\widehat{S} \le \widehat{c}_\alpha) - \mathbb{P}\left(\|N(0,\widehat{V})\|_\infty \le \widehat{c}_\alpha|\widehat{V}\right)\right| \le C^*\varepsilon + o(1).$$

This proves the validity of our test because ε can be made arbitrarily small.

In the following, we present each step in detail. We denote $\widehat{\varphi}_R^{(\ell)}$, $\widehat{\varphi}_I^{(\ell)}$ as the real and imaginary part of $\widehat{\varphi}^{(\ell)}$, respectively. According to the definition, we have that the absolute values of $\widehat{\varphi}_R^{(\ell)}$, $\widehat{\varphi}_I^{(\ell)}$ are uniformly bounded by 1, and we treat $\{\mu_b,\nu_b\}_{1\le b\le B}$ as fixed throughout the proof.

Step 1. Consider the decomposition that, for any q,μ,ν,

$$\widehat{S}(q,\mu,\nu) = S^*(q,\mu,\nu) + R_1(q,\mu,\nu) + R_2(q,\mu,\nu) + R_3(q,\mu,\nu),$$

where the remainder terms R_1, R_2 and R_3 are,

$$\begin{aligned}
R_1(q,\mu,\nu) = {} & \frac{1}{T-n-(q-1)(L-1)} \sum_{\ell=1}^{L-1}\sum_{t=1}^{n-q+1} \{\varphi^*(\mu|X_{\ell\cdot n+t+q}) - \\
& \widehat{\varphi}^{(\ell)}(\mu|X_{\ell\cdot n+t+q})\}\{\psi^*(\nu|X_{\ell\cdot n+t}) - \widehat{\psi}^{(\ell)}(\nu|X_{\ell\cdot n+t})\},\\
R_2(q,\mu,\nu) = {} & \frac{1}{T-n-(q-1)(L-1)} \sum_{\ell=1}^{L-1}\sum_{t=1}^{n-q+1} \{\exp(i\mu^\top X_{\ell\cdot n+t+q+1}) - \\
& \varphi^*(\mu|X_{\ell\cdot n+t+q})\}\{\psi^*(\nu|X_{\ell\cdot n+t}) - \widehat{\psi}^{(\ell)}(\nu|X_{\ell\cdot n+t})\},\\
R_3(q,\mu,\nu) = {} & \frac{1}{T-n-(q-1)(L-1)} \sum_{\ell=1}^{L-1}\sum_{t=1}^{n-q+1} \{\varphi^*(\mu|X_{\ell\cdot n+t+q}) - \\
& \widehat{\varphi}^{(\ell)}(\mu|X_{\ell\cdot n+t+q})\}\{\exp(i\nu^\top X_{\ell\cdot n+t-1}) - \psi^*(\nu|X_{\ell\cdot n+t})\}.
\end{aligned}$$

So it suffices to show that,

$$\max_{b\in\{1,\cdots,B\}}\max_{q\in\{2,\cdots,Q\}} \sqrt{T-n-(q-1)(L-1)}|R_m(q,\mu_b,\nu_b)| = o_p(\log^{-1/2}(T)), \tag{S19}$$

for $m = 1, 2, 3$. In the following, we show (S19) holds with $m = 1$ and $m = 2$, respectively. When $m = 3$, it can be shown similarly.

First, we show that (S19) holds when $m = 1$. Since L is fixed, it suffices to show that,

$$\max_{b\in\{1,\cdots,B\}} \max_{q\in\{2,\cdots,Q\}} \sqrt{T-n-(q-1)(L-1)}|R_{1,\ell}(q,\mu_b,\nu_b)| = o_p(\log^{-1/2}(T)), \tag{S20}$$

where $R_{1,\ell}(q,\mu_b,\nu_b)$ is of the form,

$$\frac{1}{T-n-(q-1)(L-1)}\sum_{t=1}^{n-q+1}\{\varphi^*(\mu_b|X_{\ell\cdot n+t+q})-\widehat{\varphi}^{(\ell)}(\mu_b|X_{\ell\cdot n+t+q})\}\{\psi^*(\nu_b|X_{\ell\cdot n+t})-\widehat{\psi}^{(\ell)}(\nu_b|X_{\ell\cdot n+t})\}.$$

Similarly, let φ_R^* and φ_I^* denote the real and imaginary part of φ^*, respectively. We can rewrite $R_{1,\ell}(q,\mu_b,\nu_b)$ as $R_{1,\ell}^{(1)}(q,\mu_b,\nu_b) - R_{1,\ell}^{(2)}(q,\mu_b,\nu_b) + iR_{1,\ell}^{(3)}(q,\mu_b,\nu_b) + iR_{1,\ell}^{(4)}(q,\mu_b,\nu_b)$, where

$$\begin{aligned}
R_{1,\ell}^{(1)}(q,\mu_b,\nu_b) = \frac{1}{(T-n-(q-1)(L-1))}&\sum_{t=1}^{n-q+1}\{\varphi_R^*(\mu_b|X_{\ell\cdot n+t+q})- \\
&\widehat{\varphi}_R^{(\ell)}(\mu_b|X_{\ell\cdot n+t+q})\}\{\psi_R^*(\nu_b|X_{\ell\cdot n+t})-\widehat{\psi}_R^{(\ell)}(\nu_b|X_{\ell\cdot n+t})\},\\
R_{1,\ell}^{(2)}(q,\mu_b,\nu_b) = \frac{1}{(T-n-(q-1)(L-1))}&\sum_{t=1}^{n-q+1}\{\varphi_I^*(\mu_b|X_{\ell\cdot n+t+q})- \\
&\widehat{\varphi}_I^{(\ell)}(\mu_b|X_{\ell\cdot n+t+q})\}\{\psi_I^*(\nu_b|X_{\ell\cdot n+t})-\widehat{\psi}_I^{(\ell)}(\nu_b|X_{\ell\cdot n+t})\},\\
R_{1,\ell}^{(3)}(q,\mu_b,\nu_b) = \frac{1}{(T-n-(q-1)(L-1))}&\sum_{t=1}^{n-q+1}\{\varphi_R^*(\mu_b|X_{\ell\cdot n+t+q})- \\
&\widehat{\varphi}_R^{(\ell)}(\mu_b|X_{\ell\cdot n+t+q})\}\{\psi_I^*(\nu_b|X_{\ell\cdot n+t})-\widehat{\psi}_I^{(\ell)}(\nu_b|X_{\ell\cdot n+t})\},\\
R_{1,\ell}^{(4)}(q,\mu_b,\nu_b) = \frac{1}{(T-n-(q-1)(L-1))}&\sum_{t=1}^{n-q+1}\{\varphi_I^*(\mu_b|X_{\ell\cdot n+t+q})- \\
&\widehat{\varphi}_I^{(\ell)}(\mu_b|X_{\ell\cdot n+t+q})\}\{\psi_R^*(\nu_b|X_{\ell\cdot n+t})-\widehat{\psi}_R^{(\ell)}(\nu_b|X_{\ell\cdot n+t})\}.
\end{aligned}$$

To prove (S20), it suffices to show that,

$$\max_{b\in\{1,\cdots,B\}} \max_{q\in\{2,\cdots,Q\}} \sqrt{T-n-(q-1)(L-1)}|R_{1,\ell}^{(s)}(q,\mu_b,\nu_b)| = o_p(\log^{-1/2}(T)), \tag{S21}$$

for $s = 1, 2, 3, 4$. In the following, we show that (S21) holds when $s = 1$. When $s = 2, 3, 4$, it can be shown similarly.

By Cauchy-Schwarz inequality, it suffices to show that,

$$\begin{aligned}
\max_{b\in\{1,\cdots,B\}} \max_{q\in\{2,\cdots,Q\}} \frac{1}{\sqrt{T-n-(q-1)(L-1)}}\sum_{t=1}^{n-q+1}\{\varphi_R^*(\mu_b|X_{\ell\cdot n+t})-\widehat{\varphi}_R^{(\ell)}(\mu_b|X_{\ell\cdot n+t})\}^2 \\
= o_p(\log^{-1/2}(T)),
\end{aligned} \tag{S22}$$

$$\max_{b\in\{1,\cdots,B\}}\max_{q\in\{2,\cdots,Q\}}\frac{1}{\sqrt{T-n-(q-1)(L-1)}}\sum_{t=1}^{n-q+1}\{\psi_R^*(\nu_b|X_{\ell\cdot n+t})-\widehat{\psi}_R^{(\ell)}(\nu_b|X_{\ell\cdot n+t})\}^2 \tag{S23}$$
$$=o_p(\log^{-1/2}(T)).$$

In the following, we prove (S22). The proof of (S23) is similar and is thus omitted.

By Assumption 1, $\{X_t\}_{t\ge 0}$ is exponentially β-mixing, with the coefficient $\beta(t)=O(\rho^t)$. Let $\phi_{\ell,t,b}$ denote $\varphi_R^*(\mu_b|X_{\ell\cdot n+t})-\widehat{\varphi}_R^{(\ell)}(\mu_b|X_{\ell\cdot n+t})$. We have that,

$$\max_{t,b}\mathbb{E}^{X_{\ell\cdot n+t}}\phi_{t,b}^4\le 4\max_{b\in\{1,\cdots,B\}}\int_x\{\varphi_R^*(\mu_b|x)-\widehat{\varphi}_R^{(\ell)}(\mu_b|x)\}^2\mathbb{F}(dx)\equiv\Delta, \tag{S24}$$

where the expectation $\mathbb{E}^{X_{\ell\cdot n+t}}$ is taken with respect to $X_{\ell\cdot n+t}$. Note that Δ is a random variable that depends on $\{\mu_b,\nu_b\}_{1\le b\le B}$ and $\{X_t\}_{t\in\mathcal{I}^{(\ell)}}$. By (S24), we have,

$$\max_{t,b}\mathbb{E}^{X_{\ell\cdot n+t}}(\phi_{\ell,t,b}^2-\mathbb{E}^{X_{\ell\cdot n+t}}\phi_{\ell,t,b}^2)^2\le\Delta.$$

By the boundedness assumption, we have $|\phi_{\ell,t,b}|\le 2$. Therefore, $|\phi_{\ell,t,b}^2-\mathbb{E}^{X_{\ell\cdot n+t}}\phi_{\ell,t,b}^2|\le\max\{\phi_{\ell,t,b}^2,\mathbb{E}^{X_{\ell\cdot n+t}}\phi_{\ell,t,b}^2\}\le 4$.

Similar to the idea of Step 2 of the proof for Theorem 3 using Berbee's lemma, we have that, for any integers $\tau\ge 0$ and $1<p<T/2$,

$$\mathbb{P}\left(\left|\sum_{t=1}^{n-q+1}(\phi_{\ell,t,b}^2-\mathbb{E}^{X_0}\phi_{\ell,t,b}^2)\right|\ge 6\tau\middle|\Delta\right)\le\frac{T}{p}\beta(p)+\mathbb{P}\left(\left|\sum_{t\in\mathcal{I}_r}(\phi_{\ell,t,b}^2-\mathbb{E}^{X_0}\phi_{\ell,t,b}^2)\right|\ge\tau\middle|\Delta\right)$$
$$+4\exp\left(-\frac{\tau^2/2}{(n-q+1)p\Delta+4p\tau/3}\right),$$

where $\mathcal{I}_r$ denotes the last $T-p\lfloor T/p\rfloor$ elements in the list $\{X_t\}_{t\in\mathcal{I}^{(l)}}$, and $\lfloor z\rfloor$ denotes the largest integer that is smaller than or equal to z. Suppose $\tau\ge 4p$. Noting that $|\mathcal{I}_r|\le p$, we have,

$$\mathbb{P}\left(\left|\sum_{t\in\mathcal{I}_r}(\phi_{\ell,t,b}^2-\mathbb{E}^{X_0}\phi_{\ell,t,b}^2)\right|\ge\tau\middle|\Delta\right)=0.$$

Since $\beta(t)=O(\rho^t)$, setting $p=-(c^*+3)\log(T)/\log\rho$, we obtain that $T\beta(p)/p=O(T^{-2}B^{-1})=O(B^{-1}Q^{-1}T^{-2})$, because $Q\le T$, $B=O((T)^{c_*})$. Here, the big-O notation is uniform in $b\in\{1,\cdots,B\}$, and $q\in\{2,\cdots,Q\}$. Setting $\tau=\max\{3\sqrt{\Delta Tp\log(BT)},11p\log(BT)\}$, we obtain that,

$$\frac{\tau^2}{4}\ge 2(n-q+1)p\Delta\log(BT),\quad\frac{\tau^2}{4}\ge 8p\tau\log(BT)/3,\quad\tau\ge 4p,$$

as $T \to \infty$. It follows that $\tau^2/(2(n-q+1)p\Delta + 8p\tau/3) \geq 2\log(BT)$. Therefore,

$$\max_{b\in\{1,\cdots,B\}} \max_{q\in\{2,\cdots,Q\}} \mathbb{P}\left(\left|\sum_{t=1}^{T}(\phi_{\ell,t,b}^2 - \mathbb{E}^{X_0}\phi_{\ell,t,b}^2)\right| \geq 6\tau \middle| \Delta\right) = O(B^{-1}Q^{-1}T^{-1}).$$

By Bonferroni's inequality, we obtain that,

$$\mathbb{P}\left(\max_{b\in\{1,\cdots,B\}} \max_{q\in\{2,\cdots,Q\}} \left|\sum_{t=1}^{T}(\phi_{\ell,t,b}^2 - \mathbb{E}^{X_0}\phi_{\ell,t,b}^2)\right| \geq 6\tau \middle| \Delta\right) = O(T^{-1}).$$

Therefore, with probability $1 - O(T^{-1})$, we have that,

$$\max_{b\in\{1,\cdots,B\}} \max_{q\in\{2,\cdots,Q\}} \left|\sum_{t=1}^{T}(\phi_{\ell,t,b}^2 - \mathbb{E}^{X_0}\phi_{\ell,t,b}^2)\right| = O(\sqrt{\Delta T}\log(BT), \log^2(BT)). \tag{S25}$$

Under the given condition on Q, $T-n-(q-1)(L-1)$ is proportional to T for any $q \leq Q$. Since we assume the convergence rate for $\widehat{\varphi}$ and $\widehat{\psi}$ is both $O(T^{-\kappa_0})$ for some $\kappa_0 > 1/4$, combining condition on B with (S25) yields (S22). Specifically, because of the convergence rate condition for $\widehat{\varphi}$ and $\widehat{\psi}$, we know that Δ converges to 0 in the rate of $T^{-2\kappa_0} \ll T^{-1/2}$. This shows that (S25) convergences in the rate of at least $o_p(\log^{-1/2}(T))$. In addition, such convergence rate condition also implies that $\mathbb{E}^{X_0}\phi_{\ell,t,b}^2$ convergences in the rate of $T^{-2\kappa_0}$. As a result, this term itself is negligible and (S22) is thus proved.

Next, we show that (S19) holds when $m = 2$. Similar to the proof of (S20), it suffices to show that one of the following holds,

$$\max_{q,b} \sqrt{T-n-(q-1)(L-1)}|R_{2,\ell}(q,\mu_b,\nu_b)| = o_p(\log^{-1/2}(T)), \text{ or}$$
$$\max_{q,b} \sqrt{T-n-(q-1)(L-1)}|R_{2,\ell}^{(r)}(q,\mu_b,\nu_b)| = o_p(\log^{-1/2}(T)),$$

for any $\ell = 1,\cdots,L$ and $r = 1,2,3,4$, where

$$\begin{aligned}
R_{2,\ell}(q,\mu,\nu) &= \frac{1}{T-n-(q-1)(L-1)}\sum_{t=1}^{n-q+1}\{\exp(i\mu^\top X_{\ell\cdot n+t+q-1}) \\
&\quad -\varphi^*(\mu|X_{\ell\cdot n+t+q-2})\}\{\psi^*(\nu|X_{\ell\cdot n+t}) - \widehat{\psi}^{(\ell)}(\nu|X_{\ell\cdot n+t})\}, \\
R_{2,\ell}^{(1)}(q,\mu,\nu) &= \frac{1}{T-n-(q-1)(L-1)}\sum_{t=1}^{n-q+1}\{\cos(\mu^\top X_{\ell\cdot n+t+q-1}) \\
&\quad -\varphi_R^*(\mu|X_{\ell\cdot n+t+q-2})\}\{\psi_R^*(\nu|X_{\ell\cdot n+t}) - \widehat{\psi}_R^{(\ell)}(\nu|X_{\ell\cdot n+t})\}, \\
R_{2,\ell}^{(2)}(q,\mu,\nu) &= \frac{1}{T-n-(q-1)(L-1)}\sum_{t=1}^{n-q+1}\{\sin(\mu^\top X_{\ell\cdot n+t+q-1})
\end{aligned}$$

$$-\varphi_I^*(\mu|X_{\ell\cdot n+t+q-2})\}\{\psi_I^*(\nu|X_{\ell\cdot n+t}) - \widehat{\psi}_I^{(\ell)}(\nu|X_{\ell\cdot n+t})\},$$

$$R_{2,\ell}^{(3)}(q,\mu,\nu) = \frac{1}{T-n-(q-1)(L-1)} \sum_{t=1}^{n-q+1} \{\cos(\mu^\top X_{\ell\cdot n+t+q-1})$$

$$-\varphi_R^*(\mu|X_{\ell\cdot n+t+q-2})\}\{\psi_I^*(\nu|X_{\ell\cdot n+t}) - \widehat{\psi}_I^{(\ell)}(\nu|X_{\ell\cdot n+t})\},$$

$$R_{2,\ell}^{(4)}(q,\mu,\nu) = \frac{1}{T-n-(q-1)(L-1)} \sum_{t=1}^{n-q+1} \{\sin(\mu^\top X_{\ell\cdot n+t+q-1})$$

$$-\varphi_I^*(\mu|X_{\ell\cdot n+t+q-2})\}\{\psi_R^*(\nu|X_{\ell\cdot n+t}) - \widehat{\psi}_R^{(\ell)}(\nu|X_{\ell\cdot n+t})\}.$$

In the following, we only show $\max_{q,b}\sqrt{T-n-(q-1)(L-1)}|R_{2,\ell}^{(1)}(q,\mu_b,\nu_b)| = o_p(\log^{-1/2}(T))$. The proofs of the rest of the cases are similar, and are thus omitted.

Let $\mathcal{F}_q^{(0)} = \{X_{1+n}, X_{2+n}, \cdots, X_{q-1+n}\} \cup \{X_t : t \in \bar{\mathcal{I}}^{(\ell)}\} \cup \{\mu_1,\cdots,\mu_B,\nu_1,\cdots,\nu_B\}$. Then we recursively define $\mathcal{F}_q^{(g)}$ as $\mathcal{F}_q^{(g)} = \mathcal{F}_q^{(g-1)} \cup \{X_{g+q-1+\ell n}\}$ for any $1 \le g \le n-q+1$. Let $\phi_{\ell,g,q,b}^* = \{\cos(\mu_b^\top X_{g+q-1+\ell n}) - \varphi_R^*(\mu_b|X_{g+q-2+\ell n})\}\{\psi_R^*(\nu_b|X_{g+\ell n}) - \widehat{\psi}_R^{(\ell)}(\nu_b|X_{g+\ell n})\}$. Under the Markov property, $R_{2,\ell}^{(1)}(q,\mu_b,\nu_b)$ can be rewritten as $\{T-n-(q-1)(L-1)\}^{-1}\sum_{g=1}^{n-q+1}\phi_{\ell,g,q,b}^*$, and forms a sum of martingale difference sequence with respect to the filtration $\{\sigma(\mathcal{F}_q^{(g)}) : g \ge 0\}$, where $\sigma(\mathcal{F}_q^{(g)})$ denotes the σ-algebra generated by the variables in $\mathcal{F}_q^{(g)}$. In the following, we apply concentration inequalities for martingales to bound $\max_{q,b}|R_{2,\ell}^{(1)}(q,\mu_b,\nu_b)|$.

Under the boundedness condition, we have $|\phi_{\ell,g,q,b}^*|^2 \le 4\{\psi_R^*(\nu_b|X_{g+\ell n}) - \widehat{\psi}_R^{(\ell)}(\nu_b|X_{g+\ell n})\}^2$. In addition, by the Markov property, we have that,

$$\mathbb{E}\{(\phi_{g+1,q,b}^*)^2|\sigma(\mathcal{F}_q^{(g)})\} = \mathbb{E}[\{\cos(\mu_b^\top X_{g+q-1+\ell n}) - \varphi_R^*(\mu_b|X_{g+q-2+\ell n})\}^2|X_{g+q-2+\ell n}]$$
$$\times\{\psi_R^*(\nu_b|X_{g+\ell n}) - \widehat{\psi}_R^{(\ell)}(\nu_b|X_{g+\ell n})\}^2 \le 4\{\psi_R^*(\nu_b|X_{g+\ell n}) - \widehat{\psi}_R^{(\ell)}(\nu_b|X_{g+\ell n})\}^2.$$

It follows from Theorem 2.1 of Bercu and Touati (2008) that, for any y and τ,

$$\mathbb{P}\left(\left|\sum_{g=1}^{n-q+1}\phi_{\ell,g,q,b}^*\right| \ge \tau, \sum_{g=1}^{n-q+1} 4\{\psi_R^*(\nu_b|X_{g+\ell n}) - \widehat{\psi}_R^{(\ell)}(\nu_b|X_{g+\ell n})\}^2 \le y\right) \le 2\exp\left(-\frac{\tau^2}{2y}\right).$$

Therefore, for any y and τ,

$$\mathbb{P}\left(\left|\sum_{g=1}^{n-q+1}\phi_{\ell,g,q,b}^*\right| \ge \tau, \max_{b\in\{1,\cdots,B\}}\sum_{g=1}^{n-q+1}\{\psi_R^*(\nu_b|X_{g+\ell\cdot n}) - \widehat{\psi}_R^{(\ell)}(\nu_b|X_{g+\ell\cdot n})\}^2 \le \frac{y}{4}\right) \le 2\exp\left(-\frac{\tau^2}{2y}\right).$$

By Bonferroni's inequality, we obtain that, for any y and τ,

$$\mathbb{P}\Bigg(\max_{\substack{q\in\{2,\cdots,Q\}\\ b\in\{1,\cdots,B\}}}\left|\sum_{g=1}^{n-q+1}\phi_{\ell,g,q,b}^*\right| \ge \tau, \max_{b\in\{1,\cdots,B\}}\sum_{t=1}^{n-q+1}\{\psi_R^*(\nu_b|X_{\ell\cdot n+t})$$

$$
-\widehat{\psi}_R^{(\ell)}(\nu_b|X_{\ell\cdot n+t})\}^2 \le \frac{y}{4}\Bigg) \le 2BQ\exp\left(-\frac{\tau^2}{2y}\right).
$$

Setting $y = 4\sqrt{T}$, we obtain that,

$$
\mathbb{P}\Bigg(\max_{\substack{q\in\{2,\cdots,Q\}\\ b\in\{1,\cdots,B\}}}\left|\sum_{g=1}^{n-q+1}\phi_{\ell,g,q,b}^*\right| \ge \tau, \max_{b\in\{1,\cdots,B\}}\sum_{t=1}^{n-q+1}\{\psi_R^*(\nu_b|X_{\ell\cdot n+t})
$$

$$
-\widehat{\psi}_R^{(\ell)}(\nu_b|X_{\ell\cdot n+t})\}^2 \le \sqrt{T}\Bigg) \le 2BQ\exp\left(-\frac{\tau^2}{8\sqrt{T}}\right),
$$

For any sufficiently small $\varepsilon > 0$, it follows from (S23) that,

$$
\mathbb{P}\left(\max_{\substack{q\in\{2,\cdots,Q\}\\ b\in\{1,\cdots,B\}}}\left|\sum_{g=1}^{n-q+1}\phi_{\ell,g,q,b}^*\right| \ge \tau\right) \le 2BQ\exp\left(-\frac{\tau^2}{8\sqrt{T}}\right) + o(1). \tag{S26}
$$

Setting $\tau = T^{1/4}\sqrt{8\log(BQT)}$, the right-hand-side of (S26) is $o(1)$. Under the given conditions on B and Q, we obtain that

$$
\max_{q,b}\sqrt{T-n-(q-1)(L-1)}|R_{2,\ell}^{(1)}(q,\mu_b,\nu_b)| = o_p(\log^{-1/2}(T)).
$$

Step 2. For any $0 < t < n-q+1$, define the vectors $\lambda_{R,\ell,q,t}^*, \lambda_{I,\ell,q,t}^* \in \mathbb{R}^B$, whose b-th element corresponds to the real and imaginary part of

$$
\frac{1}{\sqrt{T-n-(q-1)(L-1)}}\{\exp(i\mu_b^\top X_{\ell\cdot n+t+q-1}) - \varphi^*(\mu_b|X_{\ell\cdot n+t+q-2})\}
$$

$$
\{\exp(i\nu_b^\top X_{\ell\cdot n+t-1}) - \psi^*(\nu_b|X_{\ell\cdot n+t})\},
$$

respectively. Let $\lambda_{q,t}^*$ denote the (2B)-dimensional vector $(\lambda_{R,\ell,q,t}^{*\top}, \lambda_{I,\ell,q,t}^{*\top})^\top$. In addition, define the (2B(Q+1))-dimensional vector λ_t^* as $(\lambda_{0,t}^{*\top}, \lambda_{1,t-1}^{*\top}\mathbb{I}(t>1), \cdots, \lambda_{Q,t-Q}^{*\top}\mathbb{I}(t>Q))^\top$.

For any $1 \le g \le T-n-(q-1)(L-1)$, let $\mathcal{F}^{(0)} = \{X_1\} \cup \{\mu_1, \cdots, \mu_B, \nu_1, \cdots, \nu_B\}$ and recursively define $\mathcal{F}^{(g)} = \mathcal{F}^{(g-1)} \cup \{X_g\}$. The vector $M_{n,T} = \sum_{g=1}^{T-n-(L-1)} \lambda_g^*$ forms a sum of martingale difference sequence with respect to the filtration $\{\sigma(\mathcal{F}^{(g)}) : g \ge 0\}$. Note that $S^* = \|\sum_{g=1}^{T-n-(L-1)} \lambda_g^*\|_\infty$. In this step, we apply the high-dimensional martingale central limit theorem of Belloni and Oliveira (2018) to establish the limiting distribution of S^*. A similar result as that in Belloni and Oliveira (2018) is also given in Chernozhukov et al. (2013).

For $1 \le g \le T-n-(L-1)$, let

$$
\Sigma_g = \sum_{g=1}^{T-n-(L-1)} \mathbb{E}\left(\lambda_g^*\lambda_g^{*\top}\middle|\mathcal{F}^{(g-1)}\right), \quad \text{and} \quad V^* = \sum_{g=1}^{T-n-(L-1)} \Sigma_g.
$$

Using similar arguments as in proving (S25), we can show that, with probability $1 - O(T^{-1})$, $\|V^* - V_0\|_{\infty,\infty} = O((T)^{-1/2}\log(BT)) + O((T)^{-1}\log^2(BT))$, where $V_0 = \mathbb{E}(V^*)$. Under the given conditions on B, we have $\|V^* - V_0\|_{\infty,\infty} \le \kappa_{B,T}$, for some $\kappa_{B,T} = O((T)^{-1/2}\log(T))$, with probability $1 - O(T^{-1})$. In addition, under the boundedness assumption, all the elements in V^* and V_0 are uniformly bounded by some constant. It then follows that,

$$\mathbb{E}\|V^* - V_0\|_{\infty,\infty} \le \kappa_{B,T} + \mathbb{P}(\|V^* - V_0\|_{\infty,\infty} > \kappa_{B,T}) = O((T)^{-1/2}\log(T)).$$

By Theorem 3.1 of Belloni and Oliveira (2018), we have that, for any Borel set $\mathcal{R}$ and any $\delta > 0$,

$$\begin{aligned} &\mathbb{P}(S^* \in \mathcal{R}) \le \mathbb{P}(\|N(0,V_0)\|_\infty \in \mathcal{R}^{C\delta})| \\ &\le C\left(\frac{1}{T} + \frac{\log(BT)\log(BQ)}{\delta^2\sqrt{T}} + \frac{\log^3(BQ)}{\delta^3\sqrt{T}} + \frac{\log^3(BQ)}{\delta^3}\sum_{g=1}^{T-n-(L-1)}\mathbb{E}\|\eta_g\|_\infty^3\right), \end{aligned} \tag{S27}$$

for some constant $C > 0$.

Under the boundedness assumption, the absolute value of each element in Σ_g is uniformly bounded by $16(T - n - (q-1)(L-1))^{-1} = O(T^{-1})$. With some direct calculation, we can show that $\sum_{g=1}^{T-n-(L-1)}\mathbb{E}\|\eta_g\|_\infty^3 = O((T)^{-1/2}\log^{3/2}(BQ))$. In addition, we have $Q = O(T)$, and $B = O((T)^{c_*})$. Combining these together with (S27) yields that

$$\mathbb{P}(S^* \in \mathcal{R}) \le \mathbb{P}(\|N(0,V_0)\|_\infty \in \mathcal{R}^{C\delta})| + O(1)\left(\frac{1}{T} + \frac{\log^2(T)}{\delta^2\sqrt{T}} + \frac{\log^{9/2}(T)}{\delta^3\sqrt{T}}\right), \tag{S28}$$

where $O(1)$ denotes some positive constant.

Setting $\mathcal{R} = (z, +\infty)$ and $\delta = \varepsilon\log^{-1/2}(T)/C$, we obtain that,

$$\mathbb{P}(S^* \le z) \ge \mathbb{P}(\|N(0,V_0)\|_\infty \le z - \varepsilon\log^{-1/2}(T)) - o(1).$$

Setting $\mathcal{R} = (-\infty, z]$, we can similarly show that,

$$\mathbb{P}(S^* \le z) \le \mathbb{P}(\|N(0,V_0)\|_\infty \le z + \varepsilon\log^{-1/2}(T)) + o(1).$$

Step 3. We break this step into two parts. First, we show V_0 is a block diagonal matrix. Specifically, let V_{0,q_1,q_2} denote the $(2B)\times(2B)$ submatrix of V_0 formed by the rows in $\{2q_1B+1, 2q_1B+2, \cdots, 2(q_1+1)B\}$ and the columns in $\{2q_2B+1, 2q_2B+2, \cdots, 2(q_2+1)B\}$. For any $q_1 \ne q_2$, we show $V_{0,q_1,q_2} = O_{(2B)\times(2B)}$. Next, letting $\Sigma^{(q)}$ denote $V_{0,q,q}$, we establish an upper bound for $\max_{q\in\{2,\cdots,Q\}}\|\Sigma^{(q)} - \widehat{\Sigma}^{(q)}\|_{\infty,\infty}$. Let $\widehat{V}$ be a block diagonal matrix where the diagonal blocks are given by $\widehat{\Sigma}^{(0)}, \widehat{\Sigma}^{(1)}, \cdots, \widehat{\Sigma}^{(Q)}$, we obtain the bound for $\|V_0 - \widehat{V}\|_{\infty,\infty}$

First, let $\lambda^*_{R,q,t,b}$ and $\lambda^*_{I,q,t,b}$ denote the b-th element of $\lambda^*_{R,\ell,q,t}$ and $\lambda^*_{I,q,t}$, respectively. Each element in V_{0,q_1,q_2} equals $\mathbb{E}(\sum_t \lambda^*_{Z_1,q_1,t,b_1})(\sum_t \lambda^*_{Z_2,q_2,t,b_2})$, for some $b_1, b_2 \in \{1, \cdots, B\}$ and $Z_1, Z_2 \in \{R, I\}$. In the following, we show that,

$$\mathbb{E}\left(\sum_t \lambda^*_{R,q_1,t,b_1}\right)\left(\sum_t \lambda^*_{R,q_2,t,b_2}\right) = 0, \quad \forall q_1 \neq q_2.$$

Similarly, we can show that $\mathbb{E}(\sum_t \lambda^*_{R,q_1,t,b_1})(\sum_t \lambda^*_{I,q_2,t,b_2}) = 0$, and $\mathbb{E}(\sum_t \lambda^*_{I,q_1,t,b_1})(\sum_t \lambda^*_{I,q_2,t,b_2}) = 0$, for any $q_1 \neq q_2$.

Toward our goal, since the observations in different time series are i.i.d., it suffices to show,

$$\sum_j \mathbb{E}\left(\sum_t \lambda^*_{R,q_1,t,b_1}\right)\left(\sum_t \lambda^*_{R,q_2,t,b_2}\right) = 0, \quad \forall q_1 \neq q_2,$$

or equivalently,

$$\mathbb{E}\left(\sum_t \lambda^*_{R,q_1,0,t,b_1}\right)\left(\sum_t \lambda^*_{R,q_2,0,t,b_2}\right) = 0, \quad \forall q_1 \neq q_2, \tag{S29}$$

By definition, we have that,

$$\lambda^*_{R,q,0,t,b} = \frac{1}{\sqrt{T-n-(q-1)(L-1)}}\{\cos(\mu_b^\top X_{t+q-1+n}) - \varphi^*_R(\mu_b|X_{t+q-2+n})\}$$
$$\{\cos(\nu_b^\top X_{t-1+n}) - \psi^*_R(\nu_b|X_{t+n})\}.$$

Since $q_1 \neq q_2$, for any t_1, t_2, we have either $t_1+q_1 \neq t_2+q_2$, or $t_1 \neq t_2$. Suppose $t_1+q_1 > t_2+q_2$. Under the Markov property, we have that, for any b,

$$\mathbb{E}[\{\cos(\mu_b^\top X_{t_1+q_1-1+n}) - \varphi^*_R(\mu_b|X_{t_1+q_1-2+n})\}|\{X_j\}_{j\le t_1+q_1-2+n}] = 0.$$

Therefore, for any b_1, b_2,

$$\mathbb{E}\lambda^*_{R,q_1,0,t_1,b_1}\lambda^*_{R,q_2,0,t_2,b_2} = 0. \tag{S30}$$

Similarly, when $t_1 + q_1 < t_2 + q_2$, we can show (S30) holds as well.

Suppose $t_1 < t_2$, under H_0, we have that, for any b,

$$\mathbb{E}[\{\cos(\nu_b^\top X_{t_1-1+n}) - \varphi^*_R(\nu_b|X_{t_1+n})\}|\{X_j\}_{j\ge t_1+n}] = 0,$$

and hence (S30) holds. Similarly, when $t_1 > t_2$, we can show (S30) holds as well. This yields (S29).

Next, for any $q \in \{2, \cdots, Q\}$, we can represent $\widehat{\Sigma}^{(q)} - \Sigma^{(q)}$ by

$$\sum_{\ell=1}^{L-1} \sum_{t=1}^{n-q+1} \frac{(\lambda_{R,\ell,q,t}^\top, \lambda_{I,q,t}^\top)^\top (\lambda_{R,\ell,q,t}^\top, \lambda_{I,q,t}^\top) - (\lambda_{R,\ell,q,t}^{*\top}, \lambda_{I,q,t}^{*\top})^\top (\lambda_{R,\ell,q,t}^{*\top}, \lambda_{I,q,t}^{*\top})}{T - n - (q-1)(L-1)}. \tag{S31}$$

Using similar arguments as in Step 1 of the proof, we can show that, with probability tending to 1, the absolute value of each element in (S31) is upper bounded by $c_0^*(T)^{-c^{**}}$, for any $q \in \{2, \cdots, Q\}$ and some constants $c_0, c^* > 0$. Therefore, we obtain that, with probability tending to 1, $\max_{q \in \{2,\cdots,Q\}} \|\widehat{\Sigma}^{(q)} - \Sigma^{(q)}\|_{\infty,\infty} = O((T)^{-c^{**}})$. This completes the proof of Theorem 5. □

A.7 Proof of Theorem 6

Under the condition that $\sup_{q,\mu,\nu} S_0(q,\mu,\nu) \gg T^{-1/2} \log^{1/2}(T)$, there exist some q_0, μ_0 and ν_0, such that $S_0(q, \mu_0, \nu_0) \gg T^{-1/2} \log^{1/2}(T)$. Note that the objective function $S_0(q, \mu_0, \nu_0)$ is Lipschitz continuous in μ and ν. As such, for any μ and ν within the interval $[\mu_0 - T^{-1/2} \log^{1/2}(T), \mu_0 + T^{-1/2} \log^{1/2}(T)]$ and $[\nu_0 - T^{-1/2} \log^{1/2}(T), \nu_0 + T^{-1/2} \log^{1/2}(T)]$, we have that

$$\sup_q S_0(q, \mu, \nu) \gg T^{-1/2} \log^{1/2}(T).$$

Since each μ, ν is independently normally distributed, the probability that μ, ν falls into this interval is lower bounded by $cT^{-1/2} \log^{1/2}(T)$, for some constant $c \geq 0$. Since we randomly generate B pairs of μ, ν, the probability that at least one pair of μ, ν falls into this interval is lower bounded by

$$1 - \{1 - cT^{-1/2} \log^{1/2}(T)\}^B \geq 1 - \exp\{-cBT^{-1/2} \log^{1/2}(T)\}$$

The above probability tends to 1 under the condition that $B = \kappa_1 T^{\kappa_2}$, for some $\kappa_2 \geq 1/2$. As a result, we obtain that,

$$\max_{b \in \{1,2,\dots,B\}} \max_{q \in \{2,\cdots,Q\}} S_0(q, \mu, \nu) \gg T^{-1/2} \log^{1/2}(T)$$

Following similar arguments as in the proof of Theorem 5, we can show that,

$$\max_{b \in \{1,\cdots,B\}} \max_{q \in \{2,\cdots,Q\}} \sqrt{T - n - (q-1)(L-1)} |\widehat{S}(q, \mu_b, \nu_b) - S^*(q, \mu_b, \nu_b)| = o_p(\log^{-1/2}(T)).$$

$$\max_{b \in \{1,\cdots,B\}} \max_{q \in \{2,\cdots,Q\}} \sqrt{T - n - (q-1)(L-1)} |S_0(q, \mu_b, \nu_b) - S^*(q, \mu_b, \nu_b)| = O_p(\log^{1/2}(T)).$$

It then follows that,

$$\max_{b \in \{1,\cdots,B\}} \max_{q \in \{2,\cdots,Q\}} \sqrt{T - n - (q-1)(L-1)} |\widehat{S}(q, \mu_b, \nu_b) - S_0(q, \mu_b, \nu_b)| = O_p(\log^{1/2}(T)).$$

In addition, with some direct calculation, we have that,

$$
\begin{aligned}
&\max_{b\in\{1,\cdots,B\}} \max_{q\in\{2,\cdots,Q\}} \sqrt{T-n-(q-1)(L-1)}|\widehat{S}(q,\mu_b,\nu_b) - S_0(q,\mu_b,\nu_b)| \\
\geq &\max_{b\in\{1,\cdots,B\}} \max_{q\in\{2,\cdots,Q\}} \sqrt{T-n-(q-1)(L-1)}\Big[|S_0(q,\mu_b,\nu_b)| - |\widehat{S}(q,\mu_b,\nu_b)|\Big] \\
\geq &\max_{b\in\{1,\cdots,B\}} \max_{q\in\{2,\cdots,Q\}} \sqrt{T-n-(q-1)(L-1)}|S_0(q,\mu_b,\nu_b)| \\
&- \sqrt{T-n-(q_0+1)(L-1)}|\widehat{S}(q_0,\mu_{b_0},\nu_{b_0})|, \quad \text{for any } q_0, b_0 \\
\geq &\max_{b\in\{1,\cdots,B\}} \max_{q\in\{2,\cdots,Q\}} \sqrt{T-n-(q-1)(L-1)}|S_0(q,\mu_b,\nu_b)| \\
&- \max_{b\in\{1,\cdots,B\}} \max_{q\in\{2,\cdots,Q\}} \sqrt{T-n-(q-1)(L-1)}|\widehat{S}(q,\mu_b,\nu_b)|
\end{aligned}
$$

To summarize, we have that,

$$
\begin{aligned}
&\max_{b\in\{1,\cdots,B\}} \max_{q\in\{2,\cdots,Q\}} \sqrt{T-n-(q-1)(L-1)}|\widehat{S}(q,\mu_b,\nu_b)| \\
\geq &\max_{b\in\{1,\cdots,B\}} \max_{q\in\{2,\cdots,Q\}} \sqrt{T-n-(q-1)(L-1)}|S_0(q,\mu_b,\nu_b)| + O_p(\log^{1/2} T)
\end{aligned}
$$

Under the condition that $\max_{b\in\{1,2,\ldots,B\}} \max_{q\in\{2,\cdots,Q\}} S_0(q,\mu,\nu) \gg T^{-1/2}\log^{1/2}(T)$, we have $\widehat{S} = \max_{b\in\{1,\cdots,B\}} \max_{q\in\{2,\cdots,Q\}} \sqrt{T-n-(q-1)(L-1)}\widehat{S}(q,\mu_b,\nu_b) \gg \log^{1/2}(T)$.

Using similar arguments as in the proof of Theorem 5, we obtain that $\max_{q\in\{2,\cdots,Q\}} \|\widehat{\Sigma}^{(q)} - \Sigma^{(q)}\|_{\infty,\infty} = O(T^{-c^{**}})$, with probability tending to 1, for some constant $c^{**} > 0$. Therefore, there exist some constants $C, C' > 0$, such that $\widehat{c}_\alpha < C\log^{1/2}(B) = C'\log^{1/2}(T)$. Since $\widehat{S} \gg \log^{1/2}(T)$, we have that $\mathbb{P}(\widehat{S} > \widehat{c}_\alpha) \to 1$, as $T \to \infty$. This completes the proof of Theorem 6. □

A.8 Auxiliary Lemmas

We present a set of auxiliary lemmas that are useful for our proofs.

The first lemma establishes the approximation bound of DNN, which is used to calculate the approximation error of the mixture density network. It follows directly from Lemma 7 in Farrell et al. (2021), and its proof is omitted.

Lemma S1. *There exists a DNN class $\mathcal{F}_{DNN}$ with the ReLU activation, such that, for any $\epsilon > 0$,*

(i) $\mathcal{F}_{DNN}$ approximates $W^{\gamma,\infty}([-1,1]^d)$, in the sense that, for any $g^* \in W^{\gamma,\infty}([-1,1]^d)$, there exists a $g_\epsilon \in \mathcal{F}_{DNN}$ and $C_1 > 0$, such that $\|g_\epsilon - g^*\|_\infty \leq \epsilon$.

(ii) $H(\epsilon) \leq C_1(log(\frac{1}{\epsilon}) + 1)$ and $W(\epsilon), U(\epsilon) \leq C_1 \cdot \epsilon^{-\frac{d}{\gamma}}(\log(\frac{1}{\epsilon}) + 1)$, where H denotes the number of layers, W the number of weights, and U the total number of hidden units.

The next lemma establishes the approximation error bound for the mixture density network. This bound is affected by the approximation error in Assumption 2 through the parameter ω_1. The specific selection of ϵ in the main proof is a tradeoff between the approximation error and the Rademacher complexity.

Lemma S2. *Suppose Assumptions 2 and 3 hold. Then for any $\epsilon > 0$ and integer $G \geq 1$, there exists a set of DNN functions $\{(g_{g1}, g_{g2}, g_{g3})_{g=1}^G\}$ whose network architectures depend on ϵ such that*

(i) $\|f^* - f_T\|_\infty \leq C_2' G^{4\omega_2}\epsilon + O(G^{-\omega_1})$ *where* $f_T = \sum_{g=1}^G g_{g1}(x)\frac{1}{\sqrt{2\pi}g_{g3}(x)}e^{-\frac{(y-g_{g2}(x))^2}{2g_{g3}^2(x)}}$*, for some constant $C_2' > 0$ that is independent of ϵ;*

(ii) the number of hidden layer H_{gj}, the number of parameters W_{gj}, and the total number of hidden units U_{gj} satisfy that

- *(a)* $H_{gj} \leq C_1\{\log(G/\epsilon) + 1\}$
- *(b)* $W_{gj} \leq C_1 G^{\frac{d}{\gamma}}\, \epsilon^{-\frac{d}{\gamma}}\{\log(G/\epsilon) + 1\}$
- *(c)* $U_{gj} \leq C_1 G^{\frac{d}{\gamma}}\, \epsilon^{-\frac{d}{\gamma}}\{\log(G/\epsilon) + 1\}$

We remark that H_{gj}, W_{gj}, and U_{gj} depend on ϵ, as specified in (ii).

Proof: By Lemma S1, for any $\epsilon > 0$ and $G \geq 1$, there exists a set of DNN functions $\{(g_{g1}, g_{g2}, g_{g3})_{g=1}^G\}$, such that,

$$\|g_{g1} - \alpha_g^*\|_\infty \leq \frac{\epsilon}{G}, \quad \|g_{g2} - \mu_g^*\|_\infty \leq \frac{\epsilon}{G}, \quad \|g_{g3} - \sigma_g^*\|_\infty \leq \frac{\epsilon}{G}. \tag{S32}$$

Besides, H_{gj} and W_{gj} of the DNN functions class satisfy that $H_{gj} \leq C_1\{\log(G/\epsilon)+1\}$, $W_{gj} \leq C_1 G^{\frac{d}{\gamma}}\, \epsilon^{-\frac{d}{\gamma}}\{\log(G/\epsilon)+1\}$, and $U_{gj} \leq C_1 G^{\frac{d}{\gamma}}\, \epsilon^{-\frac{d}{\gamma}}\{\log(G/\epsilon)+1\}$. Therefore,

$$\|f^* - f_T\|_\infty = \left\| \sum_{g=1}^G \alpha_g^*(x)\frac{1}{\sqrt{2\pi}\sigma_g^*(x)}e^{-\frac{(y-\mu_g^*(x))^2}{2\sigma_g^{*2}(x)}} + O(G^{-\omega_1}) - \sum_{g=1}^G g_{g1}(x)\frac{1}{\sqrt{2\pi}g_{g3}(x)}e^{-\frac{(y-g_{g2}(x))^2}{2g_{g3}^2(x)}} \right\|_\infty$$

Since $|y| \leq 1$, $|\mu_g^*(x)| \leq C_2$, and $C_2^{-1}G^{-\omega_2} \leq \sigma_g^*(x) \leq C_2$, there exists a constant $\widetilde{C}_1$, such that

$$\left\| \frac{1}{\sqrt{2\pi}\sigma_g^*(x)}e^{-\frac{(y-\mu_g^*(x))^2}{2\sigma_g^{*2}(x)}} \right\|_\infty \leq \widetilde{C}_1 G^{\omega_2}.$$

By (S32) and since every differentiable function with bounded gradient is Lipschitz, there exists a constant $\widetilde{C}_2$, such that

$$\left\| \frac{1}{\sqrt{2\pi}\sigma_g^*(x)}e^{-\frac{(y-\mu_g^*(x))^2}{2\sigma_g^{*2}(x)}} - \frac{1}{\sqrt{2\pi}g_{g2}(x)}e^{-\frac{(y-g_{g2}(x))^2}{2g_{g3}^2(x)}} \right\|_\infty \leq \widetilde{C}_2 \frac{\epsilon G^{4\omega_2}}{G}.$$

Applying the triangle inequality, we obtain that there exists a constant $C_2' > 0$ such that,

$$\|f^* - f_T\|_\infty \le C_2' G^{4\omega_2} \epsilon + O(G^{-\omega_1})$$

This completes the proof of Lemma S2. $\square$

The next lemma connects the metric entropy between the function class of mixture density network and the deep neural network (DNN). The transition between the two metric entropies introduces a multiplier of G as the cost.

Lemma S3. *Suppose Assumptions 2 and 3 hold. Then there exists a constant $C_3 > 0$, such that*

$$\log \mathcal{T}(\delta, \mathcal{F}, \|\cdot\|_n) \le G \log \mathcal{T}\left(\frac{\delta}{C_3 G^{3/2+3\omega_2}}, \mathcal{F}_{DNN}, \|\cdot\|_n\right)$$

Proof: We first establish the Lipschitz condition. We compute the partial derivative of

$$f(y|x) = \sum_{g=1}^{G} \alpha_g(x) \frac{1}{\sqrt{2\pi}\sigma_g(x)} e^{-\frac{(y-\mu_g(x))^2}{2\sigma_g^2(x)}} \in \mathcal{F},$$

with respect to $\alpha_g, \mu_g, \sigma_g$. Since $|y| \le 1$, $0 \le \alpha_g \le 1$, $|\mu_g(x)| \le C_2$, and $C_2^{-1} G^{\omega_2} \le \sigma_g(x) \le C_2$, there exists $C_3' > 0$, which depends on A_1, A_2, A_3 and A_4, such that

$$\left|\frac{\partial f}{\partial \alpha_g}\right| = \frac{1}{\sqrt{2\pi}\sigma_g(x)} e^{-\frac{(y-\mu_g(x))^2}{2\sigma_g^2(x)}} \le C_3' G^{\omega_2}$$

$$\left|\frac{\partial f}{\partial \mu_g}\right| = \frac{|y-\mu_g(x)|}{\sigma_g^2(x)} \frac{\alpha_g}{\sqrt{2\pi}\sigma_g(x)} e^{-\frac{(y-\mu_g(x))^2}{2\sigma_g^2(x)}} \le C_3' G^{2\omega_2}$$

$$\left|\frac{\partial f}{\partial \sigma_g}\right| = \left|\frac{(y-\mu_g(x))^2}{\sigma_g^3(x)} - \frac{1}{\sigma(x)}\right| \frac{\alpha_g}{\sqrt{2\pi}\sigma_g(x)} e^{-\frac{(y-\mu_g(x))^2}{2\sigma_g^2(x)}} \le C_3' G^{3\omega_2}$$

Then, for any two neural network models $f, g \in \mathcal{F}$, there exits a constant C_3, which depends on C_3', such that

$$|f-g|^2 \le \sum_{j=1}^{G} C_3^2 G^{2\omega_2} G |h_{\alpha j}^{(f)}(x) - h_{\alpha j}^{(g)}(x)|^2$$

$$|f-g|^2 \le \sum_{j=1}^{G} C_3^2 G^{4\omega_2} G |h_{\mu j}^{(f)}(x) - h_{\mu j}^{(g)}(x)|^2$$

$$|f-g|^2 \le \sum_{j=1}^{G} C_3^2 G^{6\omega_2} G |h_{\sigma j}^{(f)}(x) - h_{\sigma j}^{(g)}(x)|^2$$

where $h^{(f)}$ and $h^{(g)}$ are for the neural network f and g, respectively, and we utilize the property that the relu, softmax and surplus functions are all 1-Lipschitz.

Next, we apply Lemma A.6 of Chernozhukov et al. (2012), and obtain that,

$$\mathcal{T}(\delta, \mathcal{F}, \|\cdot\|_n) \leq \left\{ \mathcal{T}\left(\frac{\delta}{C_3 G^{3/2+3\omega_2}}, \mathcal{F}_{\text{DNN}}, \|\cdot\|_n \right) \right\}^G. \tag{S33}$$

Taking the logarithm transformation on both sides yields that

$$\log \mathcal{T}(\delta, \mathcal{F}, \|\cdot\|_n) \leq G \log \mathcal{T}\left(\frac{\delta}{C_3 G^{3/2+3\omega_2}}, \mathcal{F}_{\text{DNN}}, \|\cdot\|_n \right). \tag{S34}$$

This completes the proof of Lemma S3. □

Since we consider the stationary time series in our setting, we cannot apply some traditional empirical inequalities, such as the Bernstein's inequality and Theorem 2.1 of Bartlett et al. (2005), developed for i.i.d. data. The next lemma relaxes the i.i.d. constraint and enables the application of those inequalities in the setting of time series with β-mixing.

Lemma S4. *Suppose there are a sequence of stationary time series, $U = (X_1, X_2, ..., X_T)$, with a common marginal distribution, and each $X_t \in \mathcal{R}^d$, $t = 1, \ldots, T$. Suppose the $\beta-$mixing coefficient of the sequence satisfies that $\beta(p) \leq c_1 e^{-c_2 p}$ for some c_1, c_2. Then there exists a sequence, $U_i^0 = (X_{ip+1}^0, X_{ip+2}^0, ..., X_{ip+p}^0)$, for $i \geq 0$, such that*

(i) U_i^0 has the same distribution as $U_i = (X_{ip+1}, X_{ip+2}, ..., X_{ip+p})$.

(ii) The sequence $\{U_{2i}^0\}_{i\geq 0}$ is i.i.d., and so is $\{U_{2i+1}^0\}_{i\geq 0}$.

(iii) For any $i \geq 0$, $\mathbb{P}(U_i \neq U_i^0) \leq \beta(p)$

(iv) $\mathbb{P}(|G_T(U) - G_T(U^0)| \neq 0) \leq p^{-1} T \beta(p)$, where $G_T(U) = \sqrt{T}\left\{\frac{1}{T}\sum_{t=1}^T f(X_{t+1}|X_t) - \mathbb{E}f\right\}$, and $G_T(U^0) = \sqrt{T}\left\{\frac{1}{T}\sum_{t=1}^T f(X_{t+1}^0|X_t^0) - \mathbb{E}f\right\}$.

Proof: The first three statements can be obtained directly from Lemma 4.1 of Dedecker and Louhichi (2002). For the last statement, we have that,

$$\mathbb{P}\left(|G_T(U) - G_T(U^0)| \neq 0\right) \leq \mathbb{P}\left(\frac{2\|f\|_\infty}{\sqrt{T}} \sum_{t=1}^T \mathbf{1}_{\{X_t \neq X_t^0\}} \neq 0 \right) \leq \frac{T}{p}\mathbb{P}\left(U_i \neq U_i^0\right) \leq \frac{T}{p}\beta(p).$$

This completes the proof of Lemma S4. □

The next lemma presents an uniform bound for the forward and the backward learner across all the choices of b. This convergence rate is important for establishing Theorem 5 and 6.

Lemma S5. *Suppose the conditions in Theorem 5 hold. Then there exists a constant $c_0 > 1/2$, such that*

$$\max_{1\le b\le B}\int_x |\widehat{\varphi}^{(\ell)}(\mu_b|x) - \varphi^*(\mu_b|x)|^2\mathbb{F}(dx) = O_p(T^{-c_0}),$$
$$\max_{1\le b\le B}\int_x |\widehat{\psi}^{(\ell)}(\nu_b|x) - \psi^*(\nu_b|x)|^2\mathbb{F}(dx) = O_p(T^{-c_0}),$$

where $\mathbb{F}$ denotes the cumulative distribution function of X_1, and $\widehat{\varphi}^{(\ell)}$ and $\widehat{\psi}^{(\ell)}$ are the bounded functions estimated by the mixture density network under Assumption 3.

Proof: By Theorem3, there exists a constants $c > 0$, such that, with probability at least $1 - O(T)$,

$$\left\|\widehat{f}^{(\ell)}_{X_{t+1}|X_t} - f^*_{X_{t+1}|X_t}\right\|_2 \le cd\left\{G^{-\omega_1} + G^{\frac{\gamma+d}{2\gamma}+4\omega_2}T^{-\frac{\gamma}{2\gamma+d}}\log^3(TG)\right\}$$

For the given $X_{(\ell-1)n+1}, ..., X_{\ell n}$, for $\widetilde{X}_{t+1}|X_t$ sampled from $\widehat{f}^{(\ell)}_{X_{t+1}|X_t}$, and a given function h bounded by some constant H_0, we have that,

$$\begin{aligned}&\frac{1}{M}\sum_{j=1}^{M} h(\widetilde{X}^{(j)}_{t+1}|X_t) - E_{f^*}h(X_{t+1}|X_t)\\ =&\frac{1}{M}\sum_{j=1}^{M} h(\widetilde{X}^{(j)}_{t+1}|X_t) - E_{\widehat{f}}h(X_{t+1}|X_t) + E_{\widehat{f}}h(X_{t+1}|X_t) - E_{f^*}h(X_{t+1}|X_t)\end{aligned} \tag{S35}$$

where E_f denotes the expectation by using f as the density function, and $E_{\widehat{f}}$ by using $\widehat{f}$ as the density. Since $|h(\widetilde{X}_{t+1}|X_t)| \le H_0$, applying the Hoeffding bound, we have that, with probability at least $1 - e^{-t^2/(2MH_0^2)} - C_{10}/M - O(T^{-1})$,

$$\sum_{j=1}^{M}\left\{h(\widetilde{X}^{(j)}_{t+1}|X_t) - E_{\widehat{f}}h(\widetilde{X}_{t+1}|X_t)\right\} \le t. \tag{S36}$$

Setting $t = M^{1/2}\log M$, and plugging (S36) into (S35), we obtain that, with probability as least $1 - M^{-1/(2H_0^2)} - C_{10}/M - O(T^{-1})$,

$$\begin{aligned}&\left|\frac{1}{M}\sum_{j=1}^{M} h(\widetilde{X}^{(j)}_{t+1}|X_t) - E_{f^*}[h(X_{t+1}|X_t)]\right|^2\\ \le&\left|\frac{1}{M^{1/2}\log M} + E_{\widehat{f}}[h(X_{t+1}|X_t)] - E_{f^*}[h(X_{t+1}|X_t)]\right|^2\\ \le&\left|\frac{1}{M^{1/2}\log M} + \int |h(y)|\frac{|\widehat{f}(y|X_t) - f^*(y|X_t)|}{f^*(y|X_t)}f^*(y|X_t)dy\right|^2\end{aligned}$$

Then by Cauchy–Schwarz inequality, we obtain that,

$$
\begin{aligned}
&\left|\frac{1}{M}\sum_{j=1}^{M} h(\widetilde{X}_{t+1}^{(j)}|X_t) - E_{f^*}[h(X_{t+1}|X_t)]\right|^2 \\
&\leq \frac{2}{M\log^2 M} + \frac{2}{C^2}\|\widehat{f}(y|X_t) - f^*(y|X_t)\|_2^2 \int |h(y)|^2 f^*(y|X_t)dy \\
&\leq \frac{2}{M\log^2 M} + \frac{2}{C^2}\|\widehat{f}(y|X_t) - f^*(y|X_t)\|_2^2 H_0^2
\end{aligned}
$$

Taking the expectation of X and $\sup_h$ on both sides, and using the result from Theorem 3, we obtain that, with probability as least $1 - (\kappa_1 T^{\kappa_2})^{-1/(2H_0^2)} - C_{10}/(\kappa_1 T^{\kappa_2}) - O(T^{-1})$,

$$
\sup_h \mathbb{E}_X \left|\frac{1}{M}\sum_{j=1}^{M} h(\widetilde{X}_{t+1}^{(j)}|X_t) - E_{f^*}[h(X_{t+1}|X_t)]\right|^2 \leq \frac{2}{M\log^2 M} + \sup_h \frac{2H_0^2}{C^2}\|\widehat{f} - f^*\|_2^2 = o(T^{-1/2}),
$$

where the last inequality holds due to the conditions of Theorem 5, where $M = \kappa_1 T^{\kappa_2}$ for some $\kappa_1 > 0$, $\kappa_2 \geq 1/2$, and the convergence rate for $\widehat{f}_{X_{t+1}|X_t}^{(\ell)}$ is $o(T^{-1/4})$. Setting the h function as the conditional characteristic functions $\widehat{\varphi}^{(\ell)}(\mu_b|x)$ and $\widehat{\psi}^{(\ell)}(\mu_b|x)$, both of which are upper bounded, then there exists a constant $c_0 \geq 1/2$, such that, with probability as least $1 - (\kappa_1 T^{\kappa_2})^{-1/(2H_0^2)} - C_{10}/(\kappa_1 T^{\kappa_2}) - O(T^{-1})$,

$$
\begin{aligned}
\max_{1\leq b\leq B} \int_x |\widehat{\varphi}^{(\ell)}(\mu_b|x) - \varphi^*(\mu_b|x)|^2 \mathbb{F}(dx) &= O_p(T^{-c_0}), \\
\max_{1\leq b\leq B} \int_x |\widehat{\psi}^{(\ell)}(\nu_b|x) - \psi^*(\nu_b|x)|^2 \mathbb{F}(dx) &= O_p(T^{-c_0}).
\end{aligned}
$$

This completes the proof of Lemma S5. □

B Additional Numerical Results

B.1 Sensitivity analysis

We conduct a sensitivity analysis to investigate the performance of the proposed test under different choices of the hyper-parameters, including the number of data chunks L, the number of pseudo samples M from the forward and backward generators, the largest number of lags Q considered in the test, as well as the number of hidden units U. Each time we vary one hyper-parameter, while we fix the rest at the default values. We consider Model 1, i.e., the VAR model, in Section 5, with $T = 500$. Figure S1 reports the percentage of times the null hypothesis is rejected out of 500 data replications at the significance level $\alpha = 0.05$. It is seen that the test

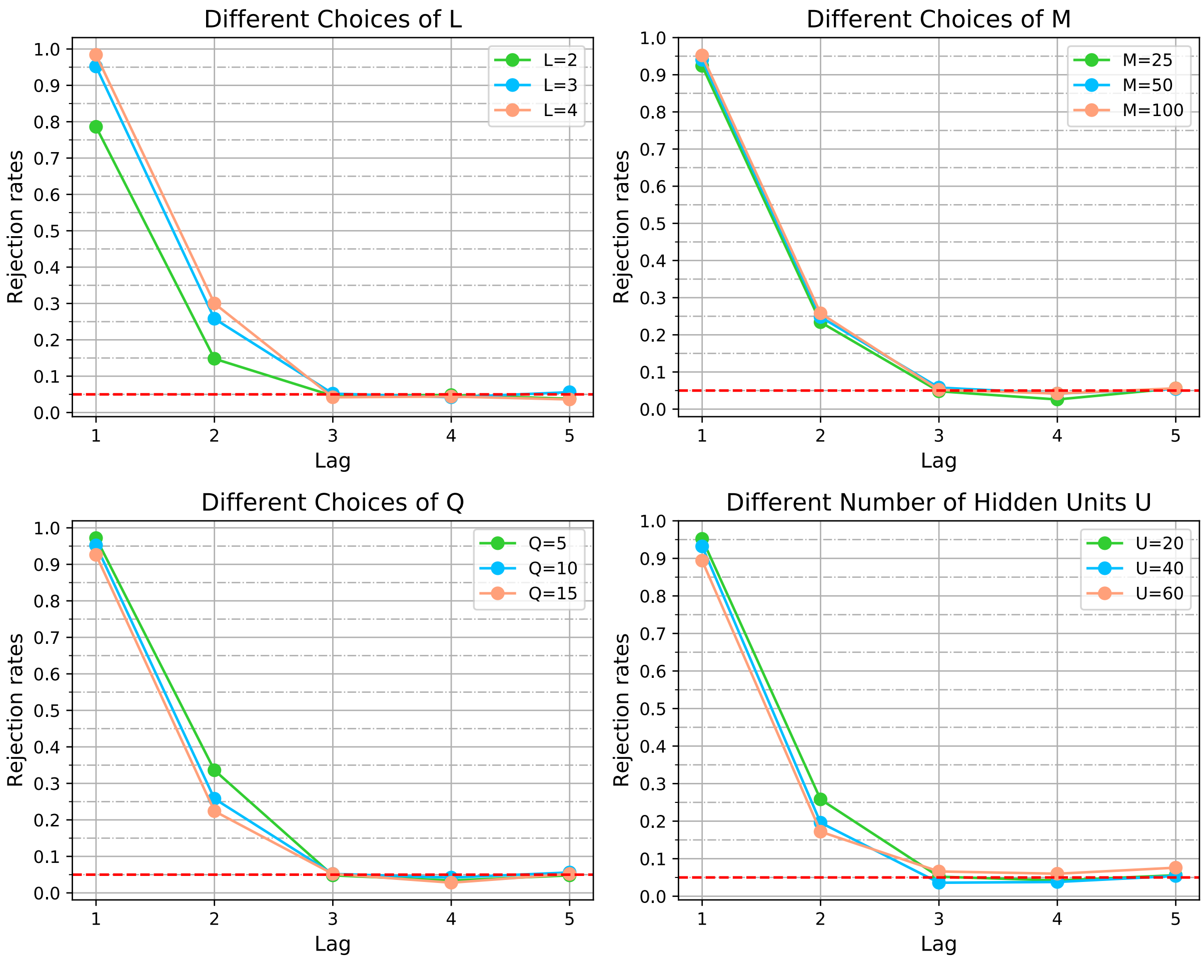

Figure S1: Sensitivity analysis: the percentage of times the null hypothesis is rejected out of 500 data replications at the significance level $\alpha = 0.05$ under difference values of the hyper-parameters L, M, Q, and U.

results are fairly stable across different values of the varying hyper-parameter, except that the power increases slightly as L increases. Overall, we conclude that the proposed test is not overly sensitive to the choice of these parameters, as long as they are in a reasonable range.

B.2 Empirical evidence of Theorem 3

Theorem 3 establishes the error bound of the MDN estimator, and is critical for the consistency of the proposed test. We carry out an additional simulation to further study the result of Theorem 3 empirically. Specifically, we consider a simple version of Model 1, i.e., a one-dimensional AR(0.5) time series model, in Section 5. Since AR(0.5) is Gaussian, MDN can exactly approximate the true density function of the data generating process, so the approximation error is

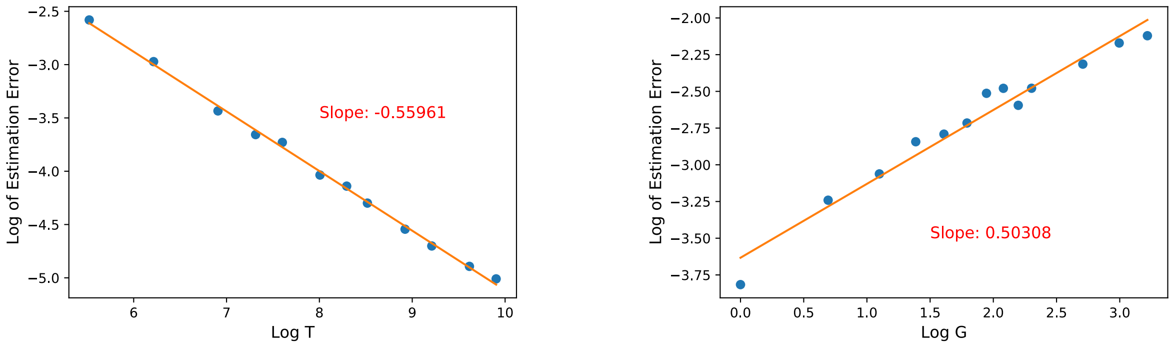

Figure S2: Average estimation error $\|\widehat{f}_{X_{t+1}|X_t} - f^*_{X_{t+1}|X_t}\|_2$ across 50 data replications, as a function of the time series length T, or the number of mixture components G, all in the log scale.

constant zero. As a result, $\|\widehat{f}_{X_{t+1}|X_t} - f^*_{X_{t+1}|X_t}\|_2$ only contains the estimation error. According to Theorem 3, $\|\widehat{f}_{X_{t+1}|X_t} - f^*_{X_{t+1}|X_t}\|_2$ should converge to zero at the rate of $O(T^{-1/2})$ when the number of mixture components G is fixed, and at the rate of $O(G^{1/2})$ when the time series length T is fixed. So we fix $G = 1$ and vary T in the first case, whereas we fix $T = 2000$ and vary G in the second case, and we record the corresponding estimation error. Figure S2 reports the average estimation error across 50 data replications when T or G varies, all in the log scale. It is seen that the slopes from the empirical results agree well with the theoretical values.

B.3 Contributing components for the proposed test

There are two key components in our proposed test: the MDN learner, and the doubly robust test statistic. We carry out additional simulations to evaluate the contributions of these components.

First, we compare the MDN learner with the local polynomial regression, in terms of the empirical convergence rate of the estimation error. We adopt Model 1, the VAR model, of Section 5, with $d = 3$. Figure S3 reports the average estimation error $\|\widehat{f}_{X_{t+1}|X_t} - f^*_{X_{t+1}|X_t}\|_2$ across 50 data replications as the time series length T varies. The left panel is based on the MDN learner, and the right panel is based the local polynomial regression. It is seen that the convergence rate of the MDN is much faster than at of the local polynomial regression.

Next, we compare our doubly robust test statistic in (6) with the test statistic in (4) that is not doubly robust, but we use the MDN learner in both cases. We again adopt Model 1, the VAR model, of Section 5, with $d = 3$. Table S1 reports the percentage of times out of 500 data replications when the null hypothesis is rejected under the significance level $\alpha = 0.05$, with the varying time series length $T = \{500, 1000, 1500\}$. The true order of the Markov model is $K = 3$. It is seen that the test statistic in (4) works well in this example as well. However, as we discuss

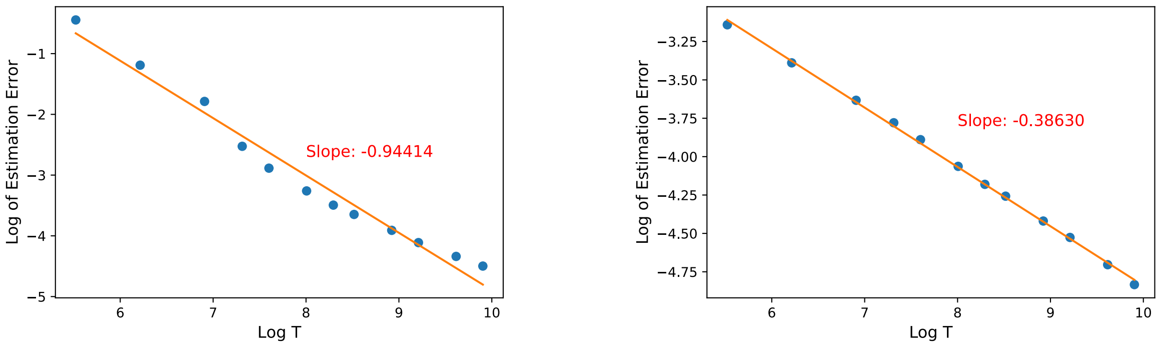

Figure S3: Average estimation error $\|\widehat{f}_{X_{t+1}|X_t} - f^*_{X_{t+1}|X_t}\|_2$ across 50 data replications, as a function of the time series length T, all in the log scale. The left panel is based on the MDN learner, and the right panel is based the local polynomial regression.

in Section 2.2, the test statistic in (4) does not have the desirable theoretical guarantee, since the bias of this test statistic has the same order of magnitude as that of the estimator of conditional characteristic function. By contrast, the doubly robust test statistic can decay to zero at a faster rate than the convergence rate of the individual estimator of CCF.

References

Anthony, M., P. L. Bartlett, P. L. Bartlett, et al. (1999). *Neural network learning: Theoretical foundations*, Volume 9. cambridge university press Cambridge.

Bartlett, P. L., O. Bousquet, and S. Mendelson (2005). Local rademacher complexities. *The Annals of Statistics 33*(4), 1497–1537.

Table S1: Percentage of times out of 500 data replications when the null hypothesis is rejected under the significance level $\alpha = 0.05$. The true order of the Markov model is $K = 3$. Two test statistics (6) and (4) are compared, while the MDN learner is used in both cases.

Order k	Test statistic	1	2	3	4	5
T = 500	(6)	0.952	0.258	0.052	0.042	0.056
	(4)	0.952	0.360	0.034	0.036	0.032
T = 1000	(6)	1.000	0.856	0.042	0.044	0.044
	(4)	1.000	0.928	0.064	0.050	0.046
T = 1500	(6)	1.000	0.992	0.060	0.058	0.048
	(4)	1.000	1.000	0.054	0.042	0.042

Belloni, A. and R. I. Oliveira (2018). A high dimensional central limit theorem for martingales, with applications to context tree models. *arXiv preprint arXiv:1809.02741*.

Berbee, H. C. P. (1979). Random walks with stationary increments and renewal theory.

Bercu, B. and A. Touati (2008). Exponential inequalities for self-normalized martingales with applications. *Ann. Appl. Probab. 18*(5), 1848–1869.

Chernozhukov, V., D. Chetverikov, M. Demirer, E. Duflo, C. Hansen, W. Newey, and J. Robins (2018). Double/debiased machine learning for treatment and structural parameters.

Chernozhukov, V., D. Chetverikov, and K. Kato (2012, 12). Gaussian approximation of suprema of empirical processes. *The Annals of Statistics*.

Chernozhukov, V., D. Chetverikov, and K. Kato (2013). Gaussian approximations and multiplier bootstrap for maxima of sums of high-dimensional random vectors. *The Annals of Statistics 41*(6), 2786–2819.

Chernozhukov, V., D. Chetverikov, and K. Kato (2017). Detailed proof of nazarov's inequality. *arXiv preprint arXiv:1711.10696*.

Dedecker, J. and S. Louhichi (2002). Maximal inequalities and empirical central limit theorems. In *Empirical process techniques for dependent data*, pp. 137–159. Springer.

Farrell, M., T. Liang, and S. Misra (2021, 01). Deep neural networks for estimation and inference. *Econometrica*, 181–213.

Mendelson, S. (2003). A few notes on statistical learning theory. In *Advanced lectures on machine learning*, pp. 1–40. Springer.

Shi, C., R. Wan, R. Song, W. Lu, and L. Leng (2020). Does the markov decision process fit the data: Testing for the markov property in sequential decision making. In *Thirty-Seventh International Conference on Machine Learning*.
